# Inducing Endogenous Cardiac Regeneration: Can Biomaterials Connect the Dots?

**DOI:** 10.3389/fbioe.2020.00126

**Published:** 2020-02-27

**Authors:** Assaf Bar, Smadar Cohen

**Affiliations:** ^1^The Avram and Stella Goldstein-Goren Department of Biotechnology Engineering, Faculty of Engineering Sciences, Ben-Gurion University of the Negev, Beersheba, Israel; ^2^Regenerative Medicine and Stem Cell Research Center, Ben-Gurion University of the Negev, Beersheba, Israel; ^3^Ilse Katz Institute for Nanoscale Science and Technology, Ben-Gurion University of the Negev, Beersheba, Israel

**Keywords:** biomaterials, cardiac patch, cardiac regeneration, drug delivery, myocardial infarction, tissue engineering

## Abstract

Heart failure (HF) after myocardial infarction (MI) due to blockage of coronary arteries is a major public health issue. MI results in massive loss of cardiac muscle due to ischemia. Unfortunately, the adult mammalian myocardium presents a low regenerative potential, leading to two main responses to injury: fibrotic scar formation and hypertrophic remodeling. To date, complete heart transplantation remains the only clinical option to restore heart function. In the last two decades, tissue engineering has emerged as a promising approach to promote cardiac regeneration. Tissue engineering aims to target processes associated with MI, including cardiomyogenesis, modulation of extracellular matrix (ECM) remodeling, and fibrosis. Tissue engineering dogmas suggest the utilization and combination of two key components: bioactive molecules and biomaterials. This chapter will present current therapeutic applications of biomaterials in cardiac regeneration and the challenges still faced ahead. The following biomaterial-based approaches will be discussed: Nano-carriers for cardiac regeneration-inducing biomolecules; corresponding matrices for their controlled release; injectable hydrogels for cell delivery and cardiac patches. The concept of combining cardiac patches with controlled release matrices will be introduced, presenting a promising strategy to promote endogenous cardiac regeneration.

## Introduction

Heart failure (HF) is a leading, growing public health problem, affecting millions of people worldwide (Laflamme and Murry, 2011). Most cases of HF are the result of a myocardial infarction (MI), defined as massive cardiac muscle death due to ischemia as a consequence of temporary or permanent blockage of blood supply to the myocardium, generally originated by thrombosis (Laflamme and Murry, 2005; Gaziano et al., 2010; Eng et al., 2014; Saleh and Ambrose, 2018). The cardiac cell death and blood vessel damage lead to extensive inflammatory responses followed by wound healing.

The human heart has only a modest capability to replace the damaged tissue and restore functionality after MI. Conversely, in the case of lower vertebrates, including some fish and amphibian species, regenerative capacity is retained throughout adult life without evidence of scarring (Matz et al., 1998; Poss et al., 2002; Cano-Martínez et al., 2010; Jopling et al., 2010). It was also shown that neonatal mammalian hearts, including mice (Porrello et al., 2011, 2013), rats, and even humans (Haubner et al., 2016), can exhibit the ability to regenerate and functionally recover in several injury models, in a limited time window immediately after birth. Genetic fate mapping in zebrafish and neonatal mice revealed that endogenous heart repair was achieved by the proliferation of pre-existing cardiomyocytes (CMs), not by the mobilization of undifferentiated precursors (Jopling et al., 2010; Kikuchi et al., 2010). However, the human adult heart consists mostly of terminally differentiated CM, excluding a small subpopulation of cardiac stem cells involved in maintaining cellular homeostasis (Torella et al., 2006). In light of these differences, following injury the damaged portions are vastly replaced by a rigid, collagenous fibrotic scar to avoid cardiac rupture (Mercola et al., 2011; Ong et al., 2018). The outcome of the wound healing process is a mechanically inferior cardiac muscle, unable to function sufficiently and mostly followed by sequential HF. Other pathologies may also impair the heart’s ability to function sufficiently and lead to HF, including valvular disease, hypertension, genetic cardiomyopathies, or aging, which cause slow functional cell loss over time.

Currently, the most common clinical intervention after MI resulting from blood vessel occlusion includes rapid re-perfusion to minimize CM death (Gerczuk and Kloner, 2012). Primary percutaneous coronary intervention (PCI) is the preferred procedure to treat narrowing or blockage of coronary arteries (stenosis). Nonetheless, a quick diagnosis (<90 min from first medical contact) of acute MI symptoms is required in order to make this strategy effective (Liem et al., 1998; Cannon et al., 2000). In the case of end-stage heart conditions, whole heart transplantation remains the only option for heart function regeneration. However, the insufficient number of donors (approximately 4000 patients on a waiting list in the US as of April 2018) and abundant post-transplantation complications limit the implementation of this strategy (Benjamin et al., 2019).

In this review, the intrinsic restraints behind endogenous cardiac regeneration following cardiac injury will be presented, defining five main target processes for therapeutic interventions. Next, cardiac regeneration strategies and limitations, namely cell-based therapies and exogenous administration of bioactive molecules will be discussed. Finally, the biomaterial-based tissue engineering approach will be introduced, focusing on current therapeutic applications of endogenous cardiac regeneration, remaining challenges and future perspectives.

### Endogenous Cardiac Regeneration Limitations

The main obstacle on the way to recovery from myocardial injury is the poor endogenous regenerative capacity of the adult mammalian heart. Cardiac regeneration is limited mainly since the majority of adult CMs do not proliferate. Shortly after birth, the cardiac tissue mechanism of growth shifts from hyperplasia to hypertrophy, meaning cell enlargement (Foglia and Poss, 2016). A major aspect of this transition is that most CMs in the mammalian myocardium withdraw from the cell cycle and grow 30–40 times in mass (Li et al., 1996). Proliferating cell populations were found to present negligible turnover rates (<1% per year and decreasing with age) (Bergmann et al., 2015). Even though there are evidences for cardiac progenitor cells (CPCs) or stem cells (CSC) residing in the adult myocardium, these subpopulations are considered minuscule (approximately 1 cell per 13,000 myocytes) (Torella et al., 2006). Even though these cell populations were observed to increase dramatically in number after MI, almost 50% exhibited a senescent phenotype, incapable of cycling and differentiating (Urbanek et al., 2005). In addition, recruitment and activation of these cells is inadequate to lead to cardiac repair due to physical barriers and lack of appropriate signaling (Ruvinov et al., 2012). Overall, these attributes result in insufficient intrinsic regenerative potential, unable to compensate for the extensive loss of heart muscle cells.

Consequently, the adult myocardium responds to MI by two main mechanisms: (a) The formation of a fibrotic scar through a wound healing process and (b) hypertrophic remodeling of the surviving myocardium (Sutton and Sharpe, 2000; Eng et al., 2014). These structural changes markedly increase the mechanical stress on the ventricular wall and promote progressive contractile dysfunction, eventually leading to HF (Ruvinov et al., 2012).

### Major Target Processes to Promote Cardiac Regeneration

Considering the poor intrinsic capability of the adult heart to properly regenerate, key hallmarks of adult heart post-MI must be overcome. Achieving effective cardiac regeneration should include tissue recovery following injury (meaning cell survival), overturn or attenuation of tissue remodeling and fibrosis, and myocardium renewal via formation of new myocardium and blood vessels. Therefore, the therapeutic strategy could target each or all the following five major processes associated with MI:

(a) Cardioprotection – prevention of resident CMs massive death (up to 1 billion cells) post-MI, by inhibition of apoptotic signaling pathways and/or induction of pro-survival signals (Garg et al., 2005; Abbate et al., 2006).
(b) Inflammation – in response to ischemia, necrotic CM signaling initiates a pro-inflammatory response, in order to remove remaining cell debris. The second phase includes an anti-inflammatory response, the purpose of which is to allow wound healing and scar formation. The shift between each of these responses is tightly regulated by multiple interactions between cellular myocardium components and the immune system. Manipulation of the time frames of the pro-/anti-inflammatory response after MI by changing the chemokine/cytokine profile or cellular responses may allow proper tissue repair to occur rather than the undesired results of an excessive inflammation, including cell death and scarring (Frangogiannis et al., 2002; Ong et al., 2018).
(c) Extracellular matrix (ECM) remodeling and cardiac fibrosis – attenuation of the fibrotic response, meaning ECM composition changes and scar formation, by altering pro-fibrotic signaling pathways. This includes inhibition of matrix metalloproteinases (MMPs) or changes in balance between MMPs and tissue residing inhibitors, in order to slow down scarring in favor of desired tissue healing (Leask, 2007; Francis Stuart et al., 2016).
(d) Angiogenesis and vascularization – formation of new blood vessels to allow appropriate nutrient supply to the ischemic regions. This could be achieved by induction of pro- angiogenesis signals, including proteins, endothelial cell (EC) recruitment, and altering inflammation and scarring (Renault and Losordo, 2007).
(e) Cardiomyogenesis – To fully restore cardiac function, it is essential that the infarct zone will be replaced by regenerated myocardium, which could involve proliferation of CPCs/CSCs, proliferation of adult CMs via induction of cell-cycle re-entry, and/or exogenous transplantation of CMs/CPCs (Mohamed et al., 2018).

## Cardiac Regeneration Strategies and Challenges

### Cell-Based Therapies

Several approaches to repair or replace injured cardiac tissue were suggested over the last two decades. One of them is the cell-based therapy, namely injection of a cell suspension. The concept is to use an engraftment of cardiac cells to reconstruct the lost muscle tissue. Injection of these suspended cells directly into the myocardium could attenuate myocardial deterioration and dysfunction.

This application requires the use of cell sources capable of yielding mature and functional CMs. Furthermore, the transplanted cells should be of autologous origin or immunotolerated by the host. Several cell sources were suggested for this purpose. CPCs/CSCs, assumed to naturally occupy the adult myocardium, are one subpopulation capable of self-renewal and differentiation. Yet, harvesting these cells requires their isolation from a myocardial biopsy, followed by cell expansion, thus limiting their availability for transplantation when treating acute MI (Torella et al., 2006).

In the last decade, human embryonic stem cells (hESCs) and induced pluripotent stem cells (iPSCs) have been considered the most attractive stem cell types as a source for *de novo*, mature CM production. Both cell types can be differentiated into CMs *in vitro* (Passier et al., 2005; Lian et al., 2013), while also having limitless cell division capability. Yet, both hESCs and iPSCs present some limitations. For instance, the origin of hESCs from blastocyst inner cell mass raises some ethical issues, along with a risk of eliciting an immune response due to their allogeneic nature (Barad et al., 2014). iPSCs are considered less immunogenic since these cells are derived from somatic cells, which potentially could be autologous to the patient. Another concern regarding the use of both types of these cells for transplantation is their pluripotency. Incomplete differentiation or ineffective isolation of the desired cell population could eventually lead to the formation of teratomas after transplantation (Nussbaum et al., 2007).

Nonetheless, this strategy alone presents very limited clinical impact due to low cell survival and retention rates inside the injected heart, resulting in only up to 10% of the delivered cells surviving 24 h after transplantation. Hence, to become clinically relevant, this kind of strategy demands the injection of an enormous number of cells (Menasché et al., 2014; Guo et al., 2017; Yanamandala et al., 2017). Besides cellular retention, cells injected within a suspension are lacking an optimal microenvironment, provided naturally by surrounding ECM. In order to reconstruct tissue organization and functionality, it is essential to supply the cells with appropriate mechanical support, topographical guidance, and proper biochemical signaling to allow desired tissue organization, differentiation, and maturation *in situ* (Frantz et al., 2010; Hubmacher and Apte, 2013; Frangogiannis, 2017).

On another note, the exact role of the injected cells in inducing cardiac regeneration is still not fully understood – do these cells act as a substitute at the injured portions of the myocardium, or perhaps, as some researchers suggest, injected cells mainly act in a paracrine manner, introducing secreted ECM and signaling molecules to surrounding host myocardium, a theory also known as “the paracrine hypothesis” (Menasché, 2008). Mesenchymal stem cell (MSC) therapy is a predominant example for this mechanism of action (Gnecchi et al., 2006; Wehman et al., 2016). MSCs, which present only limited ability to *trans*-differentiate into CMs *in vivo* (Silva et al., 2005), were shown to secret signaling molecules improving cell survival, modulating immune response, and even inducing angiogenesis (Pittenger and Martin, 2004; Thakker and Yang, 2014; Wehman et al., 2016). Although this approach was already examined in several clinical trials, evaluating different aspects of MSC delivery and their origin, it still presents major concerns limiting its efficacy. From the technical point of view, use of autologous MSCs requires their isolation and further expansion in order to be transplanted, which limits their applicability in an acute setting (Singh et al., 2016). Some clinical trials, but not all, have also highlighted safety issues, including the possibility of malignant tumor formation (Jeong et al., 2011) and paracrine proarrhythmic effects (Askar et al., 2013) post transplantation.

Recently, it was demonstrated in an ischemia mice model that the marginally improved heart function after stem cell therapy is mostly attributed to the induction of acute immune response rather than proliferation of transplanted or endogenous CMs. Vagnozzi et al. (2019) showed that the functional benefit underlying this strategy is inflammatory-based wound healing and attenuation of fibrosis, suggesting that the moderate improvement in cardiac function observed in the past corresponds better with the paracrine hypothesis.

### Bioactive Molecules

Assuming the “paracrine effect” is the engine behind cardiac regeneration, an opposing strategy to cell injection is based on the administration of bioactive signaling molecules. Three main classes of secretory factors were identified to induce cardiac regeneration, covering most of the targets specified above: growth factors (GFs) (i.e. cytokines/chemokines), non-coding RNA (i.e. microRNAs (miRNAs) and small interference RNA), and extracellular vesicles (EVs) (i.e. microvesicles and exosomes).

#### Growth Factors

Growth factors are signaling molecules, mostly proteins, which were identified to participate in various cellular processes. For example, insulin-like GF 1 (IGF-1) was previously shown to delay cellular aging and promote cell survival, while also benefit angiogenesis (Torella et al., 2004). Vascular endothelial GF (VEGF) is another cardiac-regenerating inducer; it was demonstrated to improve viability of cardiac tissue and reduce infarct size post-MI in multiple animal models through pro-angiogenic and cardiomyogenic effects (Ferrarini et al., 2006; Janavel et al., 2006). Activation of the ERBB2/ERBB4 signaling pathway by neuregulin 1 (NRG1) administration has also shown regenerative potential, driven by induction of CM proliferation (Bersell et al., 2009; D’Uva et al., 2015).

Nevertheless, the effect of systemic/local administration of GFs was inconsistent in clinical trials, showing no beneficial effects (Simons et al., 2002; Abdel-Latif et al., 2008). This could be explained by an insufficient amount of the therapeutic molecules at the target site, obligating usage of higher dosage of GFs or, alternatively, the use of a proper delivery vehicle to overcome issues of fast elimination, low protein stability, and unspecific delivery (Ruvinov et al., 2011a). Another concern is the pleiotropic functions that most cytokines have, which further encourage the use of a delivery system for their local and scheduled administration.

#### MicroRNAs

MicroRNAs are short (∼22 nucleotides long), non-coding, single stranded RNAs. Most commonly, these RNA molecules are inhibitors of protein expression, by interacting with specific mRNA in the cytoplasm, subsequently leading to mRNA cleavage or repression of translation (Lai, 2002). Numerous studies have connected miRNAs and various cardiovascular diseases, showing that miRNA expression levels were altered in MI, cardiac hypertrophy, and HF. Therefore, over the last decade miRNAs have become a therapeutic target for treatment of cardiovascular diseases (Barwari et al., 2016). For instance, Eulalio et al. (2012) performed a functional screening study to identify which human miRNAs can promote CM proliferation at neonatal stages. Two of these miRNAs, miR-199a-3p and miR-590-3p, were further demonstrated to induce cardiac regeneration in an adult mouse MI model.

Besides CM proliferation, miRNAs were shown to affect other target processes associated with MI, including miR-21, which could modulate inflammation processes and reduce CM apoptosis (Dong et al., 2009; Essandoh et al., 2016). Inhibition of miRNAs is also a possibility as demonstrated by Montgomery et al. (2011). By subcutaneous delivery of an antisense oligonucleotide they were able to inhibit miR-208a in the heart, improving cardiac function. In a preclinical trial, a catheter-based administration of a miR-92a inhibitor significantly reduced infarct size, while also excreting cardioprotective, proangiogenic, and anti-inflammatory effects (Rabea et al., 2013).

Even though miRNAs are normally detected outside the cell in body fluids, circulating miRNAs are at risk to be cleaved by RNases in these biofluids. Moreover, the strong negative charge of soluble nucleic acids results in inadequate cellular uptake (Remaut et al., 2007). Hence, it is essential to use proper vehicles to deliver miRNA, keeping it stable and protected from degradation in the extracellular environment (Chistiakov et al., 2012). Safety issues are also a concern when applying constitutive expression of miRNAs, as in the case of adeno-associated virus (AAV) delivery. Such a delivery strategy presents the risk of unwanted pathologies, such as uncontrolled heart growth (Gabisonia et al., 2019) or undesired, off-target transfection (e.g. in the liver) (Lovric et al., 2012). Therefore, it is required for miRNA delivery to be time-limited and controlled.

#### Extracellular Vesicles

Extracellular vesicles are emerging, natural carriers that could potentially deliver miRNA. These are small, membrane-enclosed vesicles that are generated and secreted by living cells. One class of EVs are exosomes, classified as double-membraned vesicles that range between 30 and 150 nm in diameter (Das and Halushka, 2015). Exosome content usually includes proteins, mRNA and miRNA.

It has been well documented that exosomes are used for communication between cells (Simons and Raposo, 2009); prominent examples can be found in cancer cells (Webber et al., 2010; Costa-Silva et al., 2015) and in the cardiovascular system (Emanueli et al., 2015). More recently, exosomes secreted by different sources of stem cells were compared to assess their potential for inducing cardiac repair in a MI model. Analysis of miRNA repertoire in exosomes secreted by iPS-derived CMs and hESCs-CMs revealed that miRNA content included some miRNAs that are known to be cardioprotective (Lee et al., 2017).

Exosomes from other cell types originating from the cardiovascular system were also shown to have potential to improve cardiac repair. For instance, monocyte-derived exosomes were shown to affect migration of ECs via exosomal transfer of miR-150, promoting angiogenesis (Zhang et al., 2010), while CPCs were demonstrated to stimulate regeneration and improve cardiac function via secretion of exosomes (Ibrahim et al., 2014; El Harane et al., 2018). The MSCs secretome was also widely investigated for its therapeutic potential, as the experience with clinical trials demonstrated only limited effects of MSC transplantation. Administration of MSC-derived exosomes contributed to cell survival, reduced inflammation, and decreased oxidative stress *in vitro* and *in vivo* (Arslan et al., 2013; Ferguson et al., 2018). Another possibility is loading exosomes with specific cargo (Luan et al., 2017), enriching them with either drugs (Sun et al., 2010), proteins (Jung et al., 2018), or miRNAs (Zhang et al., 2016). This approach could be used to manipulate exosome cargo and turn them into therapeutic agents, to be internalized by target cells and deliver desired molecules for treating different pathologies in these cells.

Even though exosome-based delivery eases the uptake by target recipient cells and is not considered toxic, it is still lacking the ability of having a long-lasting effect. Much like cytokines, exosomes have a short half-life, due to a rapid cellular internalization rate, therefore narrowing their effect to a limited time window (Kishore and Khan, 2016).

## Biomaterial-Based Tissue Engineering Approach

**FIGURE 1:**
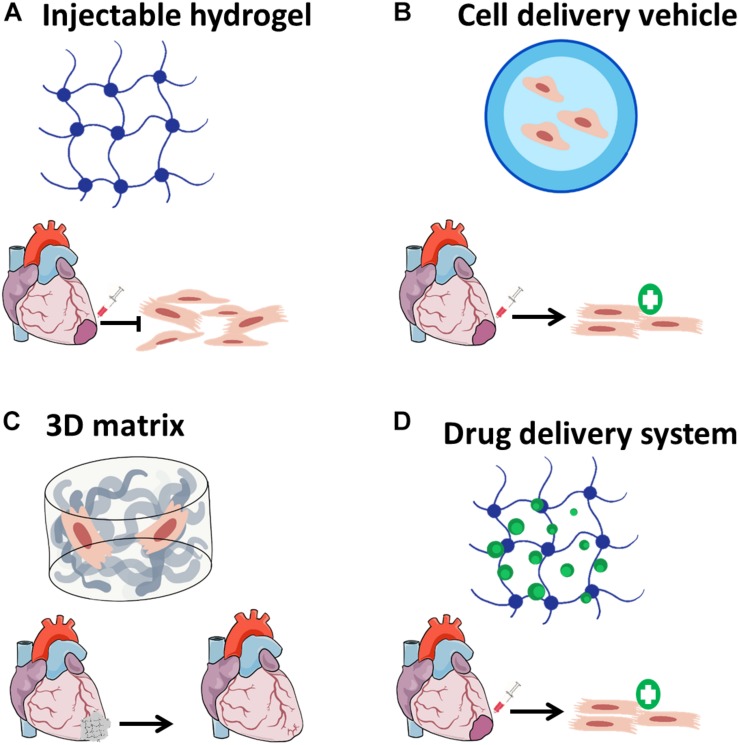
Biomaterial-based applications for cardiac tissue engineering. **(A)** Biomaterials can be injected alone into the infarcted regions in order to attenuate scar formation, **(B)** cell-delivery using injectable hydrogels can improve cell retention and survival after transplantation; **(C)** three-dimensional matrices can be fabricated with or without cells, then implanted as cardiac patches to improve cardiac function; and **(D)** biomaterials designed to release drugs and bioactive molecules may induce cardiac regeneration in a sustained effective manner.

The challenges presented by both cell-based therapies and cardiac regenerating agent delivery have set the stage for the emergence of cardiac tissue engineering (CTE). In general, TE strategies suggest using biomaterials, mostly in combination with bioactive molecules and/or a cell source for regeneration (Lanza et al., 2014). The rich repertoire of natural and synthetic polymers TE application could utilize for cardiac TE and the relevant criteria for such application were already reviewed in details elsewhere (Griffith, 2002; Christman and Lee, 2006; Chen et al., 2008; Nelson et al., 2011; Reis et al., 2016), therefore will not be addressed in the current review. Nevertheless, in the context of CTE, biomaterials can give solutions for both cell-based therapies and applications involving bioactive molecules (Figure 1).

The traditional applications of TE destinate biomaterials the role of a three-dimensional scaffold for cell retention (Langer and Vacanti, 1993). Biomaterial-based scaffolds provide the cells with needed mechanical, topographical, and biochemical stimuli, acting as ECM replacement (Yang et al., 2001). On that basis, biomaterial scaffolds were investigated in a plethora of studies for *ex vivo* cell cultivation, reviewed in Freed et al. (1994) and Shachar and Cohen (2003). These ECM properties, mimicked by biomaterials, are also of great significance when applying cell-based therapies (Leor et al., 2000).

Biomaterials, in the form of injectable hydrogel scaffolds, can be used as a vehicle to deliver cells to the target infarct zone (Yost et al., 2004; Wei et al., 2008; Ruvinov and Cohen, 2016). In parallel, the hydrogel provides the cells with the missing anchoring positions and biophysical cues, this until the cells produce their own ECM, reconstruct their microenvironments and guide their reorganization into a 3D tissue *in situ* (Dvir et al., 2005; Hubmacher and Apte, 2013).

Moreover, hydrogels embody an additional, important benefit for CTE strategies, as their injection alone into the infarct zone also reduces wall stress (Laplace law) by thickening the scar (Dai et al., 2005; Landa et al., 2008; Tsur-Gang et al., 2009). Mechanically weak, natural biomaterials are the most investigated hydrogels for this application, including collagen (McLaughlin et al., 2019), alginate (Landa et al., 2008; Sabbah et al., 2013), hyaluronic acid (HA) (Rodell et al., 2016), and decellularized myocardial matrix (Seif-Naraghi et al., 2013). In the case of the latter, it was postulated that myocardial hydrogels also have effect at the transcription level, inducing regenerative processes following their injection (Wassenaar et al., 2016). The application of mechanically strong, fully synthetic biomaterials was also beneficial (Dobner et al., 2009; Matsumura et al., 2019). Several studies, including those conducted in large animal models of MI, have shown that injection of hydrogel was effective at preventing negative tissue remodeling and improving left ventricular function post-MI, as evident by reduction in the degree of interstitial fibrosis and thickening of infarcted wall (Dobner et al., 2009; Leor et al., 2009; Rodell et al., 2016).

Biomaterials are also commonly used for the purpose of drug delivery vehicles (Koseva et al., 2015; Roy and Sahoo, 2015; Ho et al., 2016). Various polymeric materials could be used to encapsulate or entrap bioactive molecules, forming small dimension-particles (microns to sub-nano scale). Such particles enable the delivery of soluble and insoluble bioactive molecules to their target site, increasing stability, elongating drug shelf life, improving drug safety, and specificity (Roy and Sahoo, 2015).

Drug delivery platforms could also be fabricated from biomaterials, as a standalone application or incorporated with delivery vehicles. Biomaterial mechanical properties could be designed and tailored using different fabrication methods and by chemical formulation and modifications (Tibbitt and Langer, 2017; Zadpoor and Malda, 2017). Therefore, such matrices could release drugs through various mechanisms, depending on their degradation/erosion rate, exogenous triggers, or environment conditioning (Wong and Choi, 2015). This idea is an expansion of the microenvironment mimicry concept. Neighboring cells in the natural microenvironment communicate with each other by paracrine pathways, mediated by secreted proteins, small RNA species, and EVs. The ECM also acts as a reservoir system of signaling molecules (Hynes, 2009; Hinz, 2015). Incorporation of proteins and protein-binding features into biomaterials could resemble another ECM function and elicit desired cellular responses, such as cell proliferation, migration, and differentiation. Therefore, biomaterials originated (Shapira et al., 2016) or inspired (Freeman et al., 2008) by ECM components and biomechanical properties are potential candidates to perform as sophisticated drug delivery systems for spatial–temporal presentation and release of therapeutic agents, essential for endogenous tissue regeneration.

**FIGURE 2:**
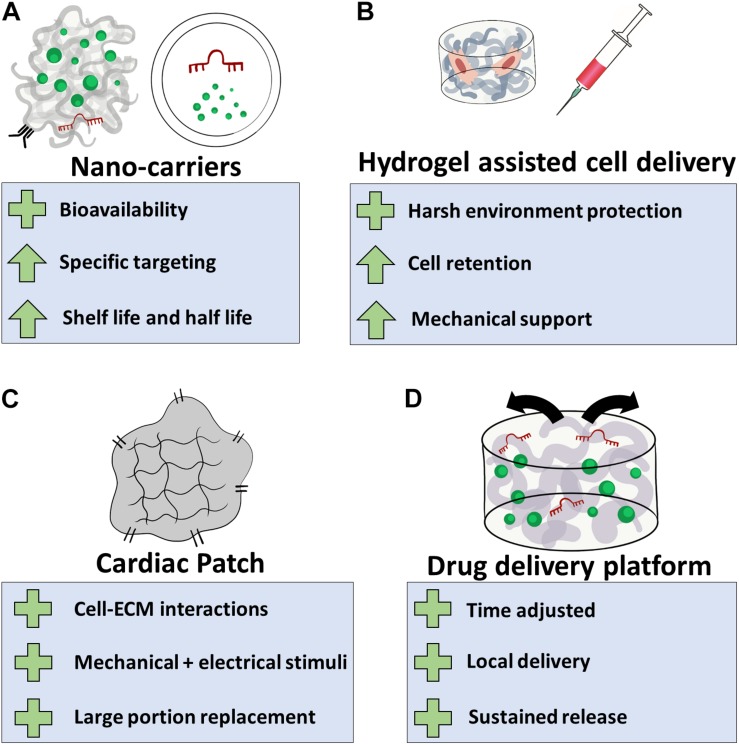
Biomaterial-based applications for cardiac regeneration. **(A)** Natural or synthetic polymers can be used as nano-carriers for the delivery of cardiac inducing agents. This will assist their bioavailability, allow specific targeting to the infarcted region or destination cell population, and increase their half-life in the tissue, eventually improving treatment efficacy; **(B)** hydrogel systems can be utilized to protect transplanted cells from the hostile post-MI microenvironment. This strategy can improve cell retention and provide the transplanted cells and the infarcted tissue the mechanical support lost as a result of massive loss of muscle tissue; **(C)** biomaterials can be used to fabricate cellular or acellular cardiac patches, providing cell with ECM interactions, mechanical and electrical stimuli. Depending on their size and mechanical properties, cardiac patches can perform as temporary or permanent replacements for damaged tissue; **(D)** regeneration-inducing agents can be encapsulated or bound to biomaterial-based delivery platforms, allowing effective release of these agents in a spatial–temporal manner. Plus sign indicates benefits of the application, arrow indicates an attribute that improves.

All these advantages are already implemented in biomaterial-based strategies meant to improve cardiac regeneration and restore functionality post cardiac injuries. In the following section, four main classes of biomaterial-based approaches will be reviewed: (a) bioactive nano-carriers; (b) hydrogel-assisted cell delivery, (c) cardiac patches, and (d) drug delivery platforms (Figure 2 and Table 1).

**TABLE 1:** Summary of biomaterials-based applications, advantages, and limitations (selected studies).

**Application**	**Biomaterial (formulation)**	**Delivered component**	**Animal model**	**Advantages**	**Limitations**	**Study**
Hydrogel-assisted cell delivery	Alginate, chitosan/β-glycerophosphate (Hydrogel)	MSCs	Rat	• Cell retention↑ • Protection from harsh microenvironment • Mechanical support↑ • Tissue integration↑	• Requires large cell quantities • Limited nutrient supply • Electrical coupling with the host	Roche et al., 2014
	Chitosan (Hydrogel)	MSCs	Rat			Liu et al., 2012
	Fibrin (Hydrogel)	Skeletal myoblasts	Rat			Christman et al., 2004
	Gelatin (Hydrogel)	MSCs	Mouse			Gottipati et al., 2019
	RGD modified, self-assembling peptide (Hydrogel)	hESCs-CMs	Mouse			Ban et al., 2014
Bioactive nano-carriers	Alginate/hyaluronan sulfate (NPs)	HGF, IGF-1	Rat	• Half-life↑ • Drug absorbance↑ • Protection from degradation • Specificity and targeting↑ • Persistence and effect↑ • Invasiveness↓	• Requires high dosages • Cytotoxicity and immunogenic response • Undesired accumulation	Ruvinov et al., 2011b
	Alginate sulfate (NPs)	miR-21	Mouse			Bejerano et al., 2018
	Peptide modified, CSC-exosomes	miRNAs, proteins	Rat			Vandergriff et al., 2018
	mESC-exosomes	miRNAs, proteins	Mouse			Khan et al., 2015a
	Liposomes	miR-199a-3p, miR-590-3p	Adult mouse			Pierluigi et al., 2017
	RGD modified, PEGylated lipid (NPs)	Puerarin	Rat			Dong et al., 2017
	Peptide modified, PEGylated liposomes	–	Mouse			Dvir et al., 2011
	MMP-specific peptide – polynorbornene amphiphiles (NPs)	–	Rat			Nguyen et al., 2015
Cardiac patches	Alginate (Scaffold)	Fetal cardiac cells	Rat	• Cell retention↑ • Protection from harsh microenvironment • Mechanical support↑ • Provides complex 3D architecture • Cell fate guidance • Replacing large tissue portions	• Requires surgery • Immunogenic response • Limited nutrient supply • Electrical coupling through large areas	Leor et al., 2000
	RGD-/HBP-modified alginate (Scaffold)	hESCs-CMs	–			Sapir et al., 2011
	Decellularized cardiac ECM–gelatin composite (Scaffold)	CPCs	Rat			Bejleri et al., 2018
	Fibrin (Scaffold)	hESCs-cardiac progenitors	Human			Menasché et al., 2018
	Fibrinogen (Scaffold)	hiPSCs-CMs	Swine			Ling et al., 2018
	Chitosan–polyaniline composite (scaffold)	–	Rat			Kapnisi et al., 2018
	Polyurethane–ECM composite (Scaffold)	–	Rat			D’Amore et al., 2016
	Fibrinogen (Scaffold)	hiPSCs-CMs	Swine			Ling et al., 2018
	Chitosan–polyaniline composite (scaffold)	–	Rat			Kapnisi et al., 2018
	Polyurethane–ECM composite (Scaffold)	–	Rat			D’Amore et al., 2016
	Porcine small intestine submucosa ECM (Scaffold)	–	Pig, ovine			Mewhort et al., 2016; Baker et al., 2019
Drug delivery platforms	Alginate-alginate sulfate (Hydrogel)	Cytokines	Rat	• Sustained drug release • Persistence and effect↑ • Protection from degradation • Local delivery • No cellular component required	• Biomaterial clearance • Immunogenic response • Inapplicable for chronic conditions	Ruvinov et al., 2011b
	Collagen (Hydrogel)	iPSC-CM-derived EVs	Rat			Liu et al., 2018
	Hyaluronic acid (Hydrogel)	miR-302/367 cluster	Rat			Wang et al., 2017
	PEG (Hydrogel)	Erythropoietin, iPSC-CMs	Rat			Chow et al., 2017

*CMs, cardiomyocytes; MSCs, mesenchymal stem cells; ESCs, embryonic stem cells; iPSCs, induced pluripotent stem cells; EVs, extracellular vesicles; NPs, nanoparticles; ECM, extracellular matrix; HGF, hepatocyte growth factor; IGF-1, insulin-like growth factor.*

## Therapeutic Applications of Biomaterials in Cardiac Regeneration

### Nano-Carriers of Bioactive Molecules

Many efforts were invested to improve the delivery of regeneration inducing agents, mainly in the means of treatment safety, efficacy, and specificity. Nano-carriers can be generally divided into two classes: nanoparticles (NPs) and natural or bio-inspired biomolecule carriers.

#### Nanoparticles

Polymer-based NPs could come in many forms, including polymeric chains enveloping therapeutic drugs, polymer–macromolecule conjugates or drugs encapsulated in polymeric micelles (Duncan, 2003). These macromolecular structures are of great pharmaceutical importance when the delivered agent has a very short half-life in circulation, or its systemic delivery is accompanied with some cytotoxicity or undesired side effects (Roy and Sahoo, 2015). The advantages of such nano-carriers were demonstrated in the case of the antioxidant drug Puerarin (PUE), an FDA approved drug for various cardiovascular diseases, including MI (Liu et al., 2016). Dong et al. (2017) developed a micelle-forming polymeric vehicle, administered intravenously and modified with Arg–Gly–Asp (RGD) peptide to increase specificity to the ischemic region. The fabricated NPs were shown to improve PUE pharmaceutical properties, including a threefold increase in half life and drug absorbance, assessed by area under the curve (AUC). Moreover, this formulation was successful at reducing infarct size in a rat MI model, presenting an enhanced effect when RGD modification was added to its surface (Dong et al., 2017).

Nanoparticles were also shown to improve protein bioavailability and decrease their toxicity due to supraphysiological dosage (Vaishya et al., 2015). For example, affinity-binding alginate can form nano-scale polymer–protein complexes (Ruvinov et al., 2016). Sulfated alginate (AlgS) was previously demonstrated to interact with heparin-binding proteins with similar affinity to heparin (Freeman et al., 2008). Ruvinov et al. (2010) showed that AlgS can co-assemble with heparin-binding proteins to form injectable NPs that protect the proteins from degradation.

Nucleic acids delivered systemically (e.g. small RNA species, mRNA, and plasmid DNA) are subjected to fast elimination and/or degradation, while also facing difficulties to enter their target cells (Remaut et al., 2007; Jones et al., 2013). Even though viral vectors are effective for delivering these agents, their application also carries significant safety issues. Thus, non-viral vehicles have emerged as a promising alternative. One of the suggested strategies is using complexes of small RNA (i.e. small interference RNA and miRNA) and plasmid DNA with calcium ions, based on electrostatic interaction between them, resulting in the formation of mildly anionic NPs (Ruvinov et al., 2015; Goldshtein et al., 2019). When these components were mixed with alginate or hyaluronan sulfate, they also spontaneously assembled into NPs mediated by ion bridges (Korin et al., 2017). The incorporation of a polymeric material to the complex enabled the addition of surface features to the resulting NPs using chemical modifications, important for targeting. These NPs were tested in order to attenuate inflammatory response and promote cardiac regeneration in a small animal MI model. In this study, miR-21 mimic was delivered in these NPs, aiming to switch macrophage phenotype to a reparative one during the innate immune system response to MI (Bejerano et al., 2018). Following intravenous administration of the NPs delivering the miR-21 mimic, the particles were shown to target macrophages in the infarcted zone and change their phenotype, resulting in reduced cell apoptosis, fibrosis, and hypertrophy.

#### Bio-Inspired Carriers

The second class of nano-carriers utilizes natural existing delivery vehicles. One example for such carriers are liposomes, closed bilayer phospholipid systems, inspired by the cellular membrane. Liposomes have been investigated for over half a century, and have been applied in very sophisticated drug delivery systems for proteins and nucleic acids, reviewed in details by Allen and Cullis (2013). In a study looking for alternatives for the use of viral vectors for miRNA mimics delivery, Pierluigi et al. (2017) assessed the efficacy of different liposomes at inducing cardiac regeneration through CM proliferation mechanism. Since liposomes lack the intrinsic ability to specifically target the heart, they used a local, intracardiac injection to deliver the liposomes straight to the infarcted area, resulting in almost 40% reduction in infarct size.

Liposomes can be targeted to the infarcted region by conjugation of specific ligands overexpressed in infarcted hearts. Such strategy was demonstrated by Dvir et al. (2011), exploiting the change in angiotensin II type 1 (AT1) receptor expression levels after hypoxia to specifically target the MI zone. They modified PEGylated liposomes’ surface by addition of a short peptide, similar on one end to angiotensin II (a ligand of AT1 receptor). Then, they showed that the NPs specifically accumulated in the left ventricular wall 7 days post-MI, but not in healthy hearts. These findings were of great significance, since they indicated not only successful targeting that increases treatment efficacy, but also passively target the heart in case of future HF (Dvir et al., 2011).

### Hydrogel-Assisted Cell Delivery

Improvement of cell retention is one of the key requirements in cell-based therapies (Menasché, 2018). Biomaterials support the transplanted cells, protecting them from the harsh environment of the infarcted region. We were among the first groups to report on a successful implantation of cardiac cell-seeded porous alginate scaffolds into infarcted rat hearts. We found that the seeded fetal rat cardiac cells retained viability within the scaffolds and within 24 h formed multicellular beating cell clusters. Following implantation of the cellular constructs into the infarcted myocardium, some of the cells appeared to differentiate into mature myocardial fibers. The graft and surrounding area were populated with a large number of newly formed blood vessels, consequently leading to attenuation in LV dilatation and improved heart function (Leor et al., 2000). An additional group demonstrated this concept using an injectable fibrin scaffold for myoblast delivery into infarcted hearts. An examination of cell location 5 weeks post injection indicated that cells injected in hydrogel were still present in the infarcted region, compared to cells suspended in BSA, which were located solely at the border zone (Christman et al., 2004). Another study demonstrated that cell viability was improved following cell administration using alginate and chitosan/β-glycerophosphate hydrogels compared to saline, showing a superior 50–60% cell retention, 24 h post transplantation (Roche et al., 2014). Since then, a variety of biomaterials and cell types were tested for cell delivery, reviewed elsewhere (O’Neill et al., 2016).

Hydrogel design criteria for cardiac cell delivery should include three key parameters: (a) mechanical stiffness of injected hydrogel; (b) physical and biochemical microenvironment suitable for encapsulated cells; and (c) duration of cell retention post transplantation. For hydrogels to withstand the mechanical demands required for myocardial applications, their stiffness modulus should be between 20 and 0.1 kPa (Reis et al., 2016). On the other hand, biomaterial elasticity could also have a role in stem cell fate determination, including differentiation (Engler et al., 2006). In case stem cells are delivered, it is mandatory to consider the use of a hydrogel system with mechanical properties resembling the myocardium in order to avoid differentiation into the wrong lineage.

Nevertheless, the microenvironment provided by the encapsulating matrix is also important in directing cell fate. Biochemical cues, including matrix-anchoring features, have a beneficial effect on cell survival and maturation. In a study aimed at enhancing engraftment of hESC-derived CMs, researchers developed a hydrogel system composed of self-assembling peptides, incorporated with RGD residue. This hydrogel not only increased cell retention in the infarcted heart, it also induced transplanted cells’ maturation and integration with the host, evident by expression of mature phenotype and formation of gap junctions with host CMs (Ban et al., 2014).

The duration of cell survival post transplantation depends on the mechanism by which the delivered cells promote regeneration. If cell transplantation aims to replace damaged tissue (e.g. CPCs, ESCs/iPSCs derived CMs), the degradation profile should allow cell integration into host myocardium (>10 days). However, if transplanted cells act by a paracrine effect (e.g. stem cells), the biomaterial should support cell survival for enough time to be effective (Levit et al., 2013). For example, the efficacy of MSC-based therapy is dependent on the number of cells surviving after transplantation (Afzal et al., 2015). Therefore, efforts have been made in order to protect cells upon delivery, using hydrogels to encapsulate MSCs, protecting them for a prolonged time from the hostile microenvironment of the ischemic region, including reactive oxygen species (Liu et al., 2012), phagocytosis, and inflammation (Gottipati et al., 2019).

### Cardiac Patches

#### Cellularized Patches

**FIGURE 3:**
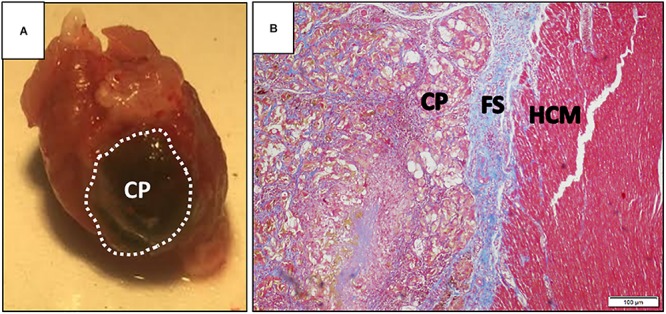
Cardiac patch after explant. **(A)** Representative image of a dissected rat heart, 30 days post-implementation of an alginate cardiac patch. The cardiac patch consists of a macroporous alginate scaffold, incorporated with magnetic nanoparticles. The construct was seeded with human embryonic stem cell-derived cardiomyocytes, and cultivated for 24 h prior to implementation on top of an infarcted rat heart. Dashed line denotes cardiac patch borders; **(B)** photomicrograph of Masson’s trichrome-stained section of the interface between implanted cardiac patch and host myocardium, 30 days post-transplantation. CP, cardiac patch; FS, fibrotic scar; HCM, host cardiac muscle. Scale bar is 100 μm. Images are courtesy of Mr. Edan Elovic.

The holy grail of CTE is the fabrication of a fully developed, three-dimensional (3D) functioning heart tissue, developed *ex vivo* and ready for transplant. In the context of cardiac malfunction, such patches could be used to replace large portions of the injured heart (Figure 3; Liau et al., 2011; Zhang et al., 2013, Zhang J. et al., 2018). Many cardiac patch designs place emphasis on providing engrafted cells with the proper microenvironment for tissue development and maturation. Besides the 3D architecture biomaterial-based matrices provided to the cells, it is essential to provide the cells with necessary ECM–cell interactions. For instance, one group suggested to use decellularized ECM of porcine origin for the fabrication of injectable hydrogels and cardiac patches, successfully demonstrating encapsulation and culture of cardiac cells (Shevach et al., 2015). Others suggested a composite of ECM components with synthetic, fibrillary, and elastic polymers, beneficial mostly at slowing the progression of scar formation and promoting angiogenesis (D’Amore et al., 2016). Similar ECM biochemical cues can be artificially added to semi-synthetic polymers. Sapir et al. (2011) suggested the covalent binding of RGD and HBP peptides to macroporous alginate scaffolds, promoting the striation and muscle fiber structure similar to that of a mature cardiac tissue. These scaffolds were further tested for their ability to promote cardiac regeneration from hESC-derived CMs, exhibiting improved functionality (Hayoun-Neeman et al., 2019).

Besides biochemical cues, anisotropic features were also shown to promote better tissue organization (Prabhakaran et al., 2011; Khan et al., 2015b; Margolis et al., 2018). Since both cardiac muscle and blood vessels are considered direction-oriented tissue constructs, different fabrication methods, including electrospinning of nanofiber structures, were investigated for influencing scaffold topography (Lutolf and Hubbell, 2005). For instance, polyurethane scaffolds were electrospun into aligned fibrous scaffolds, improving differentiation of murine ESC into CMs (Parrag et al., 2012). A more sophisticated design used a hybrid scaffold, consisted of a network of conductive nanofibers and gelatin-based hydrogel, promoting aligned and elongated CM maturation (Wu et al., 2017). Recently, our group described a method of creating a magnetically aligned, 3D tissue culture matrix for tissue engineering comprised of three distinct classes of structural anisotropy – anisotropic topographic features in the sub-micron scale, the directionality of the pore shape, and increased anisotropic stiffness in the direction of the magnetic alignment. Because magnetic forces govern the alignment phenomenon in the scaffold, all the anisotropic features shared a unidirectional structure that can synergistically benefit cultured cells (Margolis et al., 2018).

The advances in cardiac patch research are not limited to the microenvironment scale. Cardiac patch designs have demonstrated improved cellular delivery rates and feasibility to achieve a clinically relevant cardiac graft in means of size and function (Riegler et al., 2015; Shadrin et al., 2017; Ling et al., 2018). 3D bio-printing has also emerged as a strategy to assemble complex structures (Fleischer et al., 2017; Bejleri et al., 2018; Lee et al., 2019).

Other studies offer the use of exogenous stimuli to improve cardiac maturation and tissue organization *ex vivo*. After application of mechanical and electrical stimuli, iPSC-derived CMs were successfully forced to maturation and increased tissue contractility (Ruan et al., 2016). Another approach suggested to use a magnetically responsive scaffold to induce mechanical stimulation, promoting ECs organization into blood vessels (Sapir et al., 2012).

#### Acellular Patches

Even though the presence of cells contributes to cardiac recovery (either directly or in a paracrine manner), the benefits of a cardiac patch are not limited to cellular components. To date, the most investigated biomaterial used for acellular patches is decellularized ECM. Studies conducted in small (D’Amore et al., 2016) and large (Baker et al., 2019) animal models have revealed that ECM-based patches not only provide the infarcted tissue with mechanical support but also alter ventricular wall remodeling, demonstrated consistent conduction across the patch, and even promoted neovascularization. Decellularized ECM patches, originated from porcine small intestinal submucosa are already commercially available (CorMatrix^®^) (Mosala Nezhad et al., 2016). CorMatrix^®^ patches remain flexible, exhibiting no calcification after 21 months post-implant; however, the formation of new CMs was not observed in these patches, suggesting this strategy mainly has a mechanical contribution (Nelson et al., 2016). Another kind of acellular patches is designated to withstand the mechanical demands of a contracting heart. To this end, a unique auxetic pattern design was applied, allowing the patch to be stretched in multiple directions simultaneously (Kapnisi et al., 2018). Using a chitosan–polyaniline composite to fabricate a conductive scaffold (Mawad et al., 2016), the auxetic patch remained intact and attached, while maintaining left ventricular mass, presumably as a result of reduced wall stress (Kapnisi et al., 2018). Further progress was recently achieved in applying acellular patches to restore cardiac function, as a first-in-man study was completed, demonstrating the feasibility of transendocardial injection of decellularized matrix (further discussed in the section “Clinical Trials of Biomaterial-Based Applications”).

#### Challenges Facing Cardiac Patch Implementation

**FIGURE 4:**
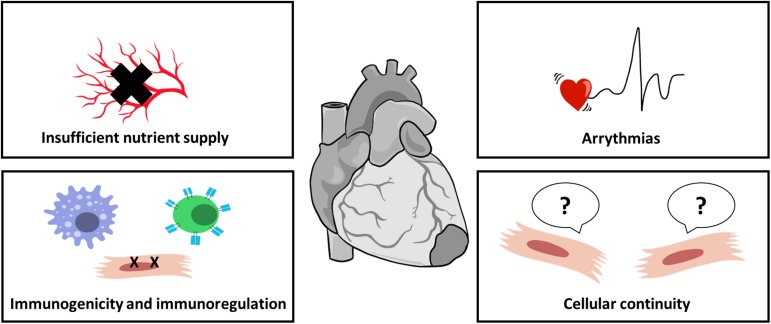
Challenges in cardiac patch implementation. Cardiac patches must integrate properly with host myocardium to properly improve cardiac function. For cell-seeded patches, sufficient nutrient supply by blood vessels is crucial for cell survival; biomaterials, as foreign objects, induce immunogenic response immediately after their introduction, impairing integration and leading to patch rejection; improper electrical coupling of the patch with host myocardium may result in arrhythmias; and cellular continuum, translated into biomechanical integrity, is also important for patch assimilation.

The successful implementation of cardiac patches as a therapeutic strategy still faces several hurdles. In order to truly restore cardiac function, the cardiac patches must assimilate with the surrounding myocardium at three levels: physical and biochemical continuity, electrophysiological communication, and nutrient supply (Figure 4).

Even though cardiac patches improve cell viability and retention, cells engrafted through such constructs or through intracardiac injection could still provoke an immunogenic response, which ultimately will lead to allograft rejection (Yanamandala et al., 2017). In addition, cardiac patch transplantation not accompanied with suppression of the host immune system will limit transplanted cell survival rate and therefore will result in failing integration (Malliaras et al., 2012).

It is also mandatory that designed scaffolds would be accessible for cell migration from areas close to the infarcted zones and allow the formation of blood vessels and nerves (Sachlos and Czernuszka, 2003; Muschler et al., 2004). Later, these networks must integrate properly with those of the host (Jackman et al., 2018).

To date, grafts still present inferior functionality, mainly in action potential conductivity (Wendel et al., 2013). Moreover, any graft transplanted would have to overcome the major restriction of integration. A graft not incorrectly integrated with the host heart tissue, both mechanically and electrically, could possibly lead to arrhythmia caused by unsynchronized electrophysiological signal transmission between the graft, the host myocardium, and the fibrotic interface in between (Chen et al., 2009). This problem was demonstrated in a study performed on primate hearts, using hESC-CM transplantation. Unlike smaller animal models, ventricular arrhythmias were observed consistently (Chong et al., 2014). This phenomenon could be related to the large distances the electrical and mechanical signals must travel, decreasing conductivity. Even in small animal models, cardiac patches were shown to fail at electrically integrating with the host, mainly due to the presence of a fibrotic scar barrier (Jackman et al., 2018). Thus, cardiac patch designs must address these limitations to be clinically relevant. Future designs must take into consideration not only the patch’s own functionality, but also its assimilation with host myocardium and synchronization over large distances.

Biomaterials, yet again, were recruited in order to overcome these hurdles. The main dogma suggests the use of porous scaffolds (Shapiro and Cohen, 1997; Dattola et al., 2019; Yang et al., 2019). Among previously mentioned advantages, such architecture also provides appropriate space and/or guiding routes for cells to penetrate the matrix. This principle could be exploited to dictate biomaterial degradation in a rate suitable for new tissue reconstruction (Bar et al., 2018). Nutrient supply and electrophysiological mediated integration could also be improved using smart biomaterial designs. For instance, VEGF containing scaffolds were designed to increase vascularization within the patch *in vivo* (Freeman and Cohen, 2009; Miyagi et al., 2011). Prevasularization of scaffolds was also suggested to improve mass transport into cardiac patches. To this end, ECM nanofibers and MSCs were used as a sheet designated to promote vascular constructs when co-cultured with ECs. They hypothesized that seeded MSCs will support the formation of microvessels and their structure through the secretion of angiogenic factors (Zhang L. et al., 2018). In order to improve electrical coupling, Shevach et al. (2014) suggested to decorate decellularized matrices with gold NPs and nanowires, presenting stronger contractile force and lowering excitation threshold.

Biomaterials were also suggested as an alternative for stitching. By using near-infrared (NIR)-stimulated gold nanowires incorporated within the scaffold, engineered tissue was positioned on the myocardium and successfully attached (Malki et al., 2018). Another scaffold design suggested the use of a microneedle array composed of poly(vinyl alcohol), integrated with CSCs encapsulated within fibrin hydrogel (Tang et al., 2018). The proposed design was strong enough to allow microneedle penetration into the ischemic region, while also enabling cell migration into the microneedle array acting mostly in a paracrine manner. Altogether, the overviewed approaches indicate that biomaterials, alongside smart scaffold designs could potentially lead to true functional integration with surrounding tissue.

### Controlled Release Platforms

Platforms of controlled, sustained release are required for applications demanding local, time adjusted, and temporary delivery of bioactive molecules. Polymeric materials, capable of forming 2D and 3D matrices, have been superior candidates for this purpose for several decades (Langer and Peppas, 1981; Hubbell, 1999; O’Neill et al., 2016). Since endogenous cardiac regeneration strategies usually aim to act locally, delivery systems impregnated within injectable hydrogels and cardiac patches are very appealing.

Wang et al. (2017) illustrated the potential of a bioactive, injectable hydrogel to induce cardiac regeneration *in situ*. They developed an injectable hydrogel, based on high-affinity interactions between two oligosaccharides, modified with HA. This hydrogel system was capable of binding cholesterol with high affinity. For the application, miR-302 mimic, an identified CM proliferating inducer, was conjugated to cholesterol and mixed within the hydrogel. The resulting hydrogel exhibited a slow, sustained release profile over 3 weeks, thus demonstrating the potential to induce cardiac proliferation in the relatively long term. Indeed, they were able to show significant clonal expansion of CMs, following miR-302 releasing hydrogel injection into infarcted hearts of Confetti mice (Wang et al., 2017).

Injectable hydrogels were also used to improve cell survival and attenuate fibrotic responses immediately after MI. Ruvinov et al. (2011b) speculated that the release of two GFs – IGF-1 (considered cardioprotective) and hepatocyte growth factor (HGF; considered anti-fibrotic) will improve cardiac regeneration by targeting two key processes in scar formation. They used injectable alginate hydrogel consisting of affinity-binding AlgS, capable of binding both GFs. The injection of the proposed hydrogel into infarcted rat hearts had positive effects: reduced myocyte apoptosis and induction of CM proliferation attenuated infarct expansion and reduced fibrosis (Ruvinov et al., 2011b).

Cell-free cardiac patches were also demonstrated to be effective at inducing endogenous cardiac regeneration, based on the “paracrine effect.” It is quite acceptable that one of the mechanisms in which cell-based therapies improve cardiac regeneration is through the secretion of cardioprotective factors, signaling surrounding cells in the infarct area. In one study, the researchers hypothesized that EVs derived from iPS or iPS-CM would have a similar effect. Therefore, they isolated these EVs *in vitro* and analyzed their miRNA content, supporting their assumptions. However, as mentioned, EVs are rapidly consumed by recipient cells, therefore their effect is time limited. In order to increase treatment efficacy, the isolated EVs were encapsulated in a cell-free collagen hydrogel, serving as a patch. The collagen hydrogel allowed a prolonged release of EVs up to 7 days *in vivo*. When the patch was applied in a rat MI model, it was able to reduce scar formation and apoptosis of CMs, while also promoting recovery of contractile functions, without any signs for arrhythmias (Liu et al., 2018). This system’s main strength is its independence on any cellular component, reducing issues of immunogenicity and concerns regarding cellular viability and retention.

## Clinical Trials of Biomaterial-Based Applications

Applications involving the use of biomaterials for cardiac regeneration have already entered clinical trials. The IK-5001 device, an injectable, bio-absorbable alginate hydrogel [also known as bioabsorbable cardiac matrix (BCM)], was tested for its safety and effectiveness for prevention of left ventricular remodeling (Bellerophon BCM LLC, ClinicalTrials.gov identifier: NCT01226563). This trial was conducted in a randomized, double-blind, controlled setup, treating 303 patients worldwide. The interventional procedure included the injection of BCM into the infarct-related artery, 2–5 days after primary PCI. Safety endpoints for this research show no significant difference from saline injections; however, the BCM treatment did not reduce LV remodeling or major cardiovascular events after a 6-month follow-up. Among the reasons for this observation, the researchers specified the large infarct sizes selected (around 30% of LV area), in which remodeling could not possibly be prevented (Rao et al., 2016).

Another acellular, alginate hydrogel formulation examined in clinical trials is the Algisyl-LVR^TM^ device (LoneStar Heart Inc., Laguna Hills, CA, United States). Algisyl-LVR^TM^ was tested for its safety and efficacy in two separate clinical trials: One aimed to quantify its effect when combined with coronary artery bypass grafting (ClinicalTrials.gov identifier: NCT0084796) and the second its employment as a method of left ventricular augmentation and restoration to treat patients with advanced chronic HF (ClinicalTrials.gov identifier: NCT01311791). In both trials the treatment included an open-heart procedure, followed by 10–15 implant injections into the left ventricular heart muscle. The first clinical trial showed remarkable improvement in cardiac function, including elevated ejection fraction (from 32 ± 8 to 47 ± 17% after 3 months), decreased end-systolic and diastolic volumes, and an increase in average wall thickness compared with control saline injections (Lee et al., 2013). In the second, randomized and controlled study, known as the AUGMENT-HF trial, effectiveness of treatment was assessed. Seventy-eight patients with dilated cardiomyopathy were selected as treatment group, and exhibited significant improvement in exercise capacity and symptoms (elevated Peak VO_2_ and longer distance in 6-min walk test) compared to control. However, these results should be considered with caution, as this trial was subjected to bias due to lack of blinding for the assignment of patients to the surgical procedure (Anker et al., 2015).

VentriGel^TM^ (Ventrix, Inc., San Diego, CA, United States), an acellular, porcine-cardiac ECM hydrogel, was also examined in a recently published phase I clinical trial (ClinicalTrials.gov identifier: NCT02305602). In this study, researchers evaluated the safety and feasibility of VentriGel^TM^. The hydrogel was delivered *trans*-endocardially within a time window between 60 days to 3 years since the first, large ST elevation MI. The outcomes of this first-in-man trial highlighted the safety and feasibility of this treatment, while also showing improvement in exercise capacity examined by a 6-min walk test, even though this study was not designed to test effectiveness of treatment (Traverse et al., 2019). Even though the collective results of clinical trials involving injection of hydrogels alone highlight the feasibility and safety of this method at improving cardiac function after MI, there is still no sufficient evidence for the capability of pristine hydrogels to promote endogenous cardiac regeneration by themselves.

CorMatrix^®^ ECM cardiac patches, which recently received FDA approval, were tested in clinical trials (CorMatrix Cardiovascular, Inc., ClinicalTrials.gov identifier: NCT02887768), claimed to promote endogenous cardiac regeneration. Pre-clinical studies with CorMatrix^®^ patches for epicardial infarct repair in a pig model supported this claim, presenting neovascularization in the interface between the infarct and the patch (Mewhort et al., 2016). Yet, in a study performed in children with congenital heart disease, there was no evidence for endogenous ingrowth of native cardiac muscle within 21 months (Nelson et al., 2016). At the present time point, clinical trials using CorMatrix patches have been designed to evaluate safety only rather than their effectiveness.

Recently, the results of a phase I clinical trial were published, determining the safety and efficacy of transplanting cardiac-committed progenitor cells derived from hESCs, using a fibrin cardiac patch (ClinicalTrials.gov identifier: NCT02057900). The fibrin cardiac patch, 20 cm^2^ in size seeded with *ex vivo* differentiated cells, was inserted inside a “pocket” under the pericardium. The results of this trial mostly demonstrated the capability to produce highly purified hESC-derived cardiac progenitor cells, without evidence of tumor formation or arrhythmias (Menasché et al., 2018). Although the feasibility to produce clinical-grade hESC-CM for transplantation was demonstrated, clinical trials assessing efficacy were not yet conducted.

## Conclusion, Limitations, and Future Perspectives

Biomaterial-based strategies offer a wide range of solutions and additives to various cardiac regeneration therapeutic approaches, making them more and more effective and clinically relevant. Biomaterials may be utilized as a vehicle for efficient delivery of regeneration-inducing small molecules and/or cells, to act as matrix guiding cell maturation (*in vitro* and *in situ*), and perform as a platform for sustained drug delivery, achieving cardiac regeneration based on the paracrine effect hypothesis. The common principle behind the use of biomaterials is their ability to function as potential mediators between the therapeutic agents and their target, while also having beneficial influence on their own.

Even though biomaterials were already tested in clinical trials for their safety, showing encouraging results and implying improvement in cardiac function, there are still some findings in pre-clinical and clinical trials that should not be overlooked. The most important issue regards foreign body response of the host to biomaterials used as medical devices. An adverse immunogenic response is common to all the biomaterial applications discussed in this review. In general, when a biomaterial, either natural or synthetic, is in contact with host tissue, it induces a response of the innate immune system, and later the adaptive immune system. Degradation products of implanted biomaterials also activate immune system components. The development of an excessive inflammatory response might severely impair the efficacy of biomaterial applications, since the endpoint of this process is fibrotic encapsulation of the device, thus minimizing its surface interplay with the host tissue and if the device is a drug delivery system, the encapsulation would affect the release profile of the drug from the device (Mariani et al., 2019). Therefore, it is extremely important to evaluate host response when applying biomaterial-based strategies for cardiac regeneration, mostly due to the adverse inflammatory response following MI. Some of the presented strategies are more susceptible to fibrotic encapsulation, mostly those involving synthetic biomaterials or containing allogeneic cellular components, thus these attributes should be taken into consideration. A possible strategy to make cellular biomaterials more tolerated is the application of immunosuppressive agents combined within the therapeutic approach (Orr et al., 2016).

Even though biomaterials are already established members in many therapeutic approaches for cardiovascular diseases, such applications have not yet reached their full potential, as there is still more room for improvements in means of mechanical stability, tolerance to immunogenic host responses, and proper integration and functional improvement with biomaterial applications. For example, the greatest challenge cardiac patches are still facing is associated with their assimilation with host myocardium. Based on the success of biomaterials to perform as drug delivery platforms and construct a highly sophisticated cardiac patch design, it is also possible that biomaterials could be the answer for that issue as well, by combining the two. For instance, incorporation of a biomaterial-based drug delivery system within a fully developed cardiac patch, acting simultaneously to treat the harmful consequences of MI while also having the potential to restore cardiac function by improving integration with the remaining, healthy cardiac tissue. Since none of the applications can fully regenerate an entire new muscle by itself, this combination could have a synergistic effect on cardiac regeneration.

## Author Contributions

AB wrote the manuscript with inputs from SC.

## Conflict of Interest

The authors declare that the research was conducted in the absence of any commercial or financial relationships that could be construed as a potential conflict of interest.

## References

[B1] Abbate A. Bussani R. Amin M. S. Vetrovec G. W. Baldi A. (2006). Acute myocardial infarction and heart failure: role of apoptosis. *Int. J. Biochem. Cell Biol.* 38 1834–1840. 10.1016/j.biocel.2006.04.010 16781883

[B2] Abdel-Latif A. Bolli R. Zuba-Surma E. K. Tleyjeh I. M. Hornung C. A. Dawn B. (2008). Granulocyte colony-stimulating factor therapy for cardiac repair after acute myocardial infarction: a systematic review and meta-analysis of randomized controlled trials. *Am. Heart J.* 156 216–226.e9. 10.1016/j.ahj.2008.03.024 18657649 PMC2597495

[B3] Afzal M. R. Samanta A. Shah Z. I. Jeevanantham V. Abdel-Latif A. Zuba-Surma E. K. (2015). Adult bone marrow cell therapy for ischemic heart disease: evidence and insights from randomized controlled trials. *Circ. Res.* 117 558–575. 10.1161/CIRCRESAHA.114.304792 26160853 PMC4553075

[B4] Allen T. M. Cullis P. R. (2013). Liposomal drug delivery systems: from concept to clinical applications. *Adv. Drug Deliv. Rev.* 65 36–48. 10.1016/j.addr.2012.09.037 23036225

[B5] Anker S. D. Coats A. J. S. Cristian G. Dragomir D. Pusineri E. Piredda M. (2015). A prospective comparison of alginate-hydrogel with standard medical therapy to determine impact on functional capacity and clinical outcomes in patients with advanced heart failure (AUGMENT-HF trial). *Eur. Heart J.* 36 2297–2309. 10.1093/eurheartj/ehv259 26082085 PMC4561351

[B6] Arslan F. Lai R. C. Smeets M. B. Akeroyd L. Choo A. Aguor E. N. E. (2013). Mesenchymal stem cell-derived exosomes increase ATP levels, decrease oxidative stress and activate PI3K/Akt pathway to enhance myocardial viability and prevent adverse remodeling after myocardial ischemia/reperfusion injury. *Stem Cell Res.* 10 301–312. 10.1016/j.scr.2013.01.002 23399448

[B7] Askar S. F. A. Ramkisoensing A. A. Atsma D. E. Schalij M. J. de Vries A. A. F. Pijnappels D. A. (2013). Engraftment patterns of human adult mesenchymal stem cells expose electrotonic and paracrine proarrhythmic mechanisms in myocardial cell cultures. *Circ. Arrhythmia Electrophysiol.* 6 380–391. 10.1161/CIRCEP.111.000215 23420831

[B8] Baker R. S. Zafar F. Kimura N. Knilans T. Osinska H. Robbins J. (2019). *In vivo* remodeling of an extracellular matrix cardiac patch in an ovine model. *Asaio J.* 65 744–752. 10.1097/MAT.0000000000000864 30153196

[B9] Ban K. Park H.-J. Kim S. Andukuri A. Cho K.-W. Hwang J. W. (2014). Cell therapy with embryonic stem cell-derived cardiomyocytes encapsulated in injectable nanomatrix gel enhances cell engraftment and promotes cardiac repair. *ACS Nano* 8 10815–10825. 10.1021/nn504617g 25210842 PMC4212793

[B10] Bar A. Ruvinov E. Cohen S. (2018). Live imaging flow bioreactor for the simulation of articular cartilage regeneration after treatment with bioactive hydrogel. *Biotechnol. Bioeng.* 115 2205–2216. 10.1002/bit.26736 29873069

[B11] Barad L. Schick R. Zeevi-Levin N. Itskovitz-Eldor J. Binah O. (2014). Human embryonic stem cells vs human induced pluripotent stem cells for cardiac repair. *Can. J. Cardiol.* 30 1279–1287. 10.1016/j.cjca.2014.06.023 25442431

[B12] Barwari T. Joshi A. Mayr M. (2016). MicroRNAs in cardiovascular disease. *J. Am. Coll. Cardiol.* 68 2577–2584. 10.1016/j.jacc.2016.09.945 27931616

[B13] Bejerano T. Etzion S. Elyagon S. Etzion Y. Cohen S. (2018). Nanoparticle delivery of miRNA-21 mimic to cardiac macrophages improves myocardial remodeling after myocardial infarction. *Nano Lett.* 18 5885–5891. 10.1021/acs.nanolett.8b02578 30141949

[B14] Bejleri D. Streeter B. W. Nachlas A. L. Y. Brown M. E. Gaetani R. Christman K. L. (2018). A bioprinted cardiac patch composed of cardiac-specific extracellular matrix and progenitor cells for heart repair. *Adv. Healthc. Mater.* 7:1800672. 10.1002/adhm.201800672 30379414 PMC6521871

[B15] Benjamin E. J. Muntner P. Bittencourt M. S. (2019). Heart disease and stroke statistics-2019 update: a report From the American Heart Association. *Circulation* 139 e56–e528. 10.1161/CIR.0000000000000659 30700139

[B16] Bergmann O. Zdunek S. Felker A. Salehpour M. Alkass K. Bernard S. (2015). Dynamics of cell generation and turnover in the human heart. *Cell* 161 1566–1575. 10.1016/j.cell.2015.05.026 26073943

[B17] Bersell K. Arab S. Haring B. Kühn B. (2009). Neuregulin1/ErbB4 signaling induces cardiomyocyte proliferation and repair of heart injury. *Cell* 138 257–270. 10.1016/j.cell.2009.04.060 19632177

[B18] Cannon C. P. Gibson C. M. Lambrew C. T. Shoultz D. A. Levy D. French W. J. (2000). Relationship of symptom-onset-to-balloon time and door-to-balloon time with mortality in patients undergoing angioplasty for acute myocardial infarction. *JAMA* 283 2941–2947. 10865271 10.1001/jama.283.22.2941

[B19] Cano-Martínez A. Vargas-González A. Guarner-Lans V. Prado-Zayago E. Leon-Oleda M. Nieto-Lima B. (2010). Functional and structural regeneration in the axolotl heart (*Ambystoma mexicanum*) after partial ventricular amputation. *Arch. Cardiol. Mex.* 80 79–86. 21147570

[B20] Chen H.-S. V. Kim C. Mercola M. (2009). Electrophysiological challenges of cell-based myocardial repair. *Circulation* 120 2496–2508. 10.1161/circulationaha.107.751412 20008740 PMC3293934

[B21] Chen Q.-Z. Harding S. E. Ali N. N. Lyon A. R. Boccaccini A. R. (2008). Biomaterials in cardiac tissue engineering: ten years of research survey. *Mater. Sci. Eng. R Rep.* 59 1–37. 10.1016/j.mser.2007.08.001

[B22] Chistiakov D. A. Sobenin I. A. Orekhov A. N. (2012). Strategies to deliver microRNAs as potential therapeutics in the treatment of cardiovascular pathology. *Drug Deliv.* 19 392–405. 10.3109/10717544.2012.738436 23173580

[B23] Chong J. J. H. Yang X. Don C. W. Minami E. Liu Y.-W. Weyers J. J. (2014). Human embryonic-stem-cell-derived cardiomyocytes regenerate non-human primate hearts. *Nature* 510 273–277. 10.1038/nature13233 24776797 PMC4154594

[B24] Chow A. Stuckey D. J. Kidher E. Rocco M. Jabbour R. J. Mansfield C. A. (2017). Human induced pluripotent stem cell-derived cardiomyocyte encapsulating bioactive hydrogels improve rat heart function post myocardial infarction. *Stem Cell Rep.* 9 1415–1422. 10.1016/j.stemcr.2017.09.003 28988988 PMC5830963

[B25] Christman K. L. Lee R. J. (2006). Biomaterials for the treatment of myocardial infarction. *J. Am. Coll. Cardiol.* 48 907–913. 10.1016/j.jacc.2006.06.005 16949479

[B26] Christman K. L. Vardanian A. J. Fang Q. Sievers R. E. Fok H. H. Lee R. J. (2004). Injectable fibrin scaffold improves cell transplant survival, reduces infarct expansion, and induces neovasculature formation in ischemic myocardium. *J. Am. Coll. Cardiol.* 44 654–660. 10.1016/j.jacc.2004.04.040 15358036

[B27] Costa-Silva B. Aiello N. M. Ocean A. J. Singh S. Zhang H. Thakur B. K. (2015). Pancreatic cancer exosomes initiate pre-metastatic niche formation in the liver. *Nat. Cell Biol.* 17 816–826. 25985394 10.1038/ncb3169 PMC5769922

[B28] Dai W. Wold L. E. Dow J. S. Kloner R. A. (2005). Thickening of the infarcted wall by collagen injection improves left ventricular function in rats: a novel approach to preserve cardiac function after myocardial infarction. *J. Am. Coll. Cardiol.* 46 714–719. 10.1016/j.jacc.2005.04.056 16098441

[B29] D’Amore A. Yoshizumi T. Luketich S. K. Wolf M. T. Gu X. Cammarata M. (2016). Bi-layered polyurethane – Extracellular matrix cardiac patch improves ischemic ventricular wall remodeling in a rat model. *Biomaterials* 107 1–14. 10.1016/j.biomaterials.2016.07.039 27579776

[B30] Das S. Halushka M. K. (2015). Extracellular vesicle microRNA transfer in cardiovascular disease. *Cardiovasc. Pathol.* 24 199–206. 10.1016/j.carpath.2015.04.007 25958013

[B31] Dattola E. Parrotta E. I. Scalise S. Perozziello G. Limongi T. Candeloro P. (2019). Development of 3D PVA scaffolds for cardiac tissue engineering and cell screening applications. *RSC Adv.* 9 4246–4257. 10.1039/c8ra08187e PMC9060459 35520194

[B32] Dobner S. Bezuidenhout D. Govender P. Zilla P. Davies N. (2009). A synthetic non-degradable polyethylene glycol hydrogel retards adverse post-infarct left ventricular remodeling. *J. Card. Fail.* 15 629–636. 10.1016/j.cardfail.2009.03.003 19700140

[B33] Dong S. Cheng Y. Yang J. Li J. Liu X. Wang X. (2009). MicroRNA expression signature and the role of microRNA-21 in the early phase of acute myocardial infarction. *J. Biol. Chem.* 284 29514–29525. 10.1074/jbc.M109.027896 19706597 PMC2785585

[B34] Dong Z. Guo J. Xing X. Zhang X. Du Y. Lu Q. (2017). RGD modified and PEGylated lipid nanoparticles loaded with puerarin: formulation, characterization and protective effects on acute myocardial ischemia model. *Biomed. Pharmacother.* 89 297–304. 10.1016/j.biopha.2017.02.029 28236703

[B35] Duncan R. (2003). The dawning era of polymer therapeutics. *Nat. Rev. Drug Discov.* 2 347–360. 10.1038/nrd1088 12750738

[B36] D’Uva G. Aharonov A. Lauriola M. Kain D. Yahalom-Ronen Y. Carvalho S. (2015). ERBB2 triggers mammalian heart regeneration by promoting cardiomyocyte dedifferentiation and proliferation. *Nat. Cell Biol.* 17 627–638. 10.1038/ncb3149 25848746

[B37] Dvir T. Bauer M. Schroeder A. Tsui J. H. Anderson D. G. Langer R. (2011). Nanoparticles targeting the infarcted heart. *Nano Lett.* 11 4411–4414. 10.1021/nl2025882 21899318 PMC3192253

[B38] Dvir T. Tsur-Gang O. Cohen S. (2005). “Designer” scaffolds for tissue engineering and regeneration. *Isr. J. Chem.* 45 487–494. 10.1560/378J-XMB1-NAKF-YKQ1

[B39] El Harane N. Kervadec A. Bellamy V. Pidial L. Neametalla H. J. Perier M.-C. (2018). Acellular therapeutic approach for heart failure: *in vitro* production of extracellular vesicles from human cardiovascular progenitors. *Eur. Heart J.* 39 1835–1847. 10.1093/eurheartj/ehy012 29420830 PMC6251654

[B40] Emanueli C. Shearn A. I. U. Angelini G. D. Sahoo S. (2015). Exosomes and exosomal miRNAs in cardiovascular protection and repair. *Vascul. Pharmacol.* 71 24–30. 10.1016/j.vph.2015.02.008 25869502 PMC4838026

[B41] Eng G. Lee B. W. Radisic M. Vunjak-Novakovic G. (2014). “Chapter 38 - Cardiac Tissue Engineering,” in *Principles of Tissue Engeenering*, 4th Edn, eds Lanza R. Langer R. Vacanti J. (Boston, MA: Academic Press), 771–792. 10.1016/B978-0-12-398358-9.00038-0

[B42] Engler A. J. Sen S. Sweeney H. L. Discher D. E. (2006). Matrix elasticity directs stem cell lineage specification. *Cell* 126 677–689. 10.1016/j.cell.2006.06.044 16923388

[B43] Essandoh K. Li Y. Huo J. Fan G.-C. (2016). MiRNA-mediated macrophage polarization and its potential role in the regulation of inflammatory response. *Shock* 46 122–131. 10.1097/SHK.0000000000000604 26954942 PMC4949115

[B44] Eulalio A. Mano M. Dal Ferro M. Zentilin L. Sinagra G. Zacchigna S. (2012). Functional screening identifies miRNAs inducing cardiac regeneration. *Nature* 492 376–381. 10.1038/nature11739 23222520

[B45] Ferguson S. W. Wang J. Lee C. J. Liu M. Neelamegham S. Canty J. M. (2018). The microRNA regulatory landscape of MSC-derived exosomes: a systems view. *Sci. Rep.* 8:1419. 10.1038/s41598-018-19581-x 29362496 PMC5780426

[B46] Ferrarini M. Arsic N. Recchia F. A. Zentilin L. Zacchigna S. Xu X. (2006). Adeno-associated virus-mediated transduction of VEGF165 improves cardiac tissue viability and functional recovery after permanent coronary occlusion in conscious dogs. *Circ. Res.* 98 954–961. 10.1161/01.res.0000217342.83731.89 16543500

[B47] Fleischer S. Feiner R. Dvir T. (2017). Cutting-edge platforms in cardiac tissue engineering. *Curr. Opin. Biotechnol.* 47 23–29. 10.1016/j.copbio.2017.05.008 28578251

[B48] Foglia M. J. Poss K. D. (2016). Building and re-building the heart by cardiomyocyte proliferation. *Development* 143 729–740. 10.1242/dev.132910 26932668 PMC4813344

[B49] Francis Stuart S. D. De Jesus N. M. Lindsey M. L. Ripplinger C. M. (2016). The crossroads of inflammation, fibrosis, and arrhythmia following myocardial infarction. *J. Mol. Cell. Cardiol.* 91 114–122. 10.1016/j.yjmcc.2015.12.024 26739214 PMC4764395

[B50] Frangogiannis N. G. (2017). The extracellular matrix in myocardial injury, repair, and remodeling. *J. Clin. Invest.* 127 1600–1612. 10.1172/JCI87491 28459429 PMC5409799

[B51] Frangogiannis N. G. Smith C. W. Entman M. L. (2002). The inflammatory response in myocardial infarction. *Cardiovasc. Res.* 53 31–47. 10.1016/s0008-6363(01)00434-5 11744011

[B52] Frantz C. Stewart K. M. Weaver V. M. (2010). The extracellular matrix at a glance. *J. Cell Sci.* 123 4195–4200. 10.1242/jcs.023820 21123617 PMC2995612

[B53] Freed L. E. Vunjak-Novakovic G. Biron R. J. Eagles D. B. Lesnoy D. C. Barlow S. K. (1994). Biodegradable polymer scaffolds for tissue engineering. *Biotechnology* 12 689–693. 10.1038/nbt0794-689 7764913

[B54] Freeman I. Cohen S. (2009). The influence of the sequential delivery of angiogenic factors from affinity-binding alginate scaffolds on vascularization. *Biomaterials* 30 2122–2131. 10.1016/j.biomaterials.2008.12.057 19152972

[B55] Freeman I. Kedem A. Cohen S. (2008). The effect of sulfation of alginate hydrogels on the specific binding and controlled release of heparin-binding proteins. *Biomaterials* 29 3260–3268. 10.1016/j.biomaterials.2008.04.025 18462788

[B56] Gabisonia K. Prosdocimo G. Aquaro G. D. Carlucci L. Zentilin L. Secco I. (2019). MicroRNA therapy stimulates uncontrolled cardiac repair after myocardial infarction in pigs. *Nature* 569 418–422. 10.1038/s41586-019-1191-6 31068698 PMC6768803

[B57] Garg S. Narula J. Chandrashekhar Y. (2005). Apoptosis and heart failure: clinical relevance and therapeutic target. *J. Mol. Cell. Cardiol.* 38 73–79. 10.1016/j.yjmcc.2004.11.006 15623423

[B58] Gaziano T. A. Bitton A. Anand S. Abrahams-Gessel S. Murphy A. (2010). Growing epidemic of coronary heart disease in low-and middle-income countries. *Curr. Probl. Cardiol.* 35 72–115. 10.1016/j.cpcardiol.2009.10.002 20109979 PMC2864143

[B59] Gerczuk P. Z. Kloner R. A. (2012). An update on cardioprotection. *J. Am. Coll. Cardiol.* 59 969–978. 10.1016/j.jacc.2011.07.054 22402067

[B60] Gnecchi M. He H. Noiseux N. Liang O. D. Zhang L. Morello F. (2006). Evidence supporting paracrine hypothesis for Akt-modified mesenchymal stem cell-mediated cardiac protection and functional improvement. *FASEB J.* 20 661–669. 10.1096/fj.05-5211com 16581974

[B61] Goldshtein M. Shamir S. Vinogradov E. Monsonego A. Cohen S. (2019). Co-assembled Ca^2+^ alginate-sulfate nanoparticles for intracellular plasmid DNA delivery. *Mol. Ther. Nucleic Acids* 16 378–390. 10.1016/j.omtn.2019.03.006 31003172 PMC6475713

[B62] Gottipati A. Chelvarajan L. Peng H. Kong R. Cahall C. F. Li C. (2019). Gelatin based polymer cell coating improves bone marrow-derived cell retention in the heart after myocardial infarction. *Stem Cell Rev. Rep.* 15 404–414. 10.1007/s12015-018-9870-5 30644039 PMC6535106

[B63] Griffith L. G. (2002). Emerging design principles in biomaterials and scaffolds for tissue engineering. *Ann. N. Y. Acad. Sci.* 961 83–95. 10.1111/j.1749-6632.2002.tb03056.x 12081872

[B64] Guo Y. Wysoczynski M. Nong Y. Tomlin A. Zhu X. Gumpert A. M. (2017). Repeated doses of cardiac mesenchymal cells are therapeutically superior to a single dose in mice with old myocardial infarction. *Basic Res. Cardiol.* 112:18. 10.1007/s00395-017-0606-5 28210871 PMC5655998

[B65] Haubner B. J. Schneider J. Schweigmann U. Schuetz T. Dichtl W. Velik-Salchner C. (2016). Functional recovery of a human neonatal heart after severe myocardial infarction. *Circ. Res.* 118 216–221. 10.1161/CIRCRESAHA.115.307017 26659640

[B66] Hayoun-Neeman D. Korover N. Etzion S. Ofir R. Lichtenstein R. G. Cohen S. (2019). Exploring peptide-functionalized alginate scaffolds for engineering cardiac tissue from human embryonic stem cell-derived cardiomyocytes in serum-free medium. *Polym. Adv. Technol.* 30 2493–2505. 10.1002/pat.4602

[B67] Hinz B. (2015). The extracellular matrix and transforming growth factor-β1: tale of a strained relationship. *Matrix Biol.* 47 54–65. 10.1016/j.matbio.2015.05.006 25960420

[B68] Ho W. Zhang X.-Q. Xu X. (2016). Biomaterials in siRNA delivery: a comprehensive review. *Adv. Healthc. Mater.* 5 2715–2731. 10.1002/adhm.201600418 27700013

[B69] Hubbell J. A. (1999). Bioactive biomaterials. *Curr. Opin. Biotechnol.* 10 123–129. 10.1016/S0958-1669(99)80021-4 10209141

[B70] Hubmacher D. Apte S. S. (2013). The biology of the extracellular matrix: novel insights. *Curr. Opin. Rheumatol.* 25 65–70. 10.1097/BOR.0b013e32835b137b 23143224 PMC3560377

[B71] Hynes R. O. (2009). The extracellular matrix: not just pretty fibrils. *Science* 326 1216–1219. 10.1126/science.1176009 19965464 PMC3536535

[B72] Ibrahim A. G.-E. Cheng K. Marbán E. (2014). Exosomes as critical agents of cardiac regeneration triggered by cell therapy. *Stem Cell Rep.* 2 606–619. 10.1016/j.stemcr.2014.04.006 24936449 PMC4050492

[B73] Jackman C. P. Ganapathi A. M. Asfour H. Qian Y. Allen B. W. Li Y. (2018). Engineered cardiac tissue patch maintains structural and electrical properties after epicardial implantation. *Biomaterials* 159 48–58. 10.1016/j.biomaterials.2018.01.002 29309993 PMC5801076

[B74] Janavel G. V. Crottogini A. Meckert P. C. Cuniberti L. Mele A. Papouchado M. (2006). Plasmid-mediated VEGF gene transfer induces cardiomyogenesis and reduces myocardial infarct size in sheep. *Gene Ther.* 13 1133–1142. 10.1038/sj.gt.3302708 16572192

[B75] Jeong J.-O. Han J. W. Kim J.-M. Cho H.-J. Park C. Lee N. (2011). Malignant tumor formation after transplantation of short-term cultured bone marrow mesenchymal stem cells in experimental myocardial infarction and diabetic neuropathy. *Circ. Res.* 108 1340–1347. 10.1161/CIRCRESAHA.110.239848 21493893 PMC3109741

[B76] Jones C. H. Chen C.-K. Ravikrishnan A. Rane S. Pfeifer B. A. (2013). Overcoming nonviral gene delivery barriers: perspective and future. *Mol. Pharm.* 10 4082–4098. 10.1021/mp400467x 24093932 PMC5232591

[B77] Jopling C. Sleep E. Raya M. Martí M. Raya A. Izpisúa Belmonte J. C. (2010). Zebrafish heart regeneration occurs by cardiomyocyte dedifferentiation and proliferation. *Nature* 464 606–609. 10.1038/nature08899 20336145 PMC2846535

[B78] Jung K. O. Jo H. Yu J. H. Gambhir S. S. Pratx G. (2018). Development and MPI tracking of novel hypoxia-targeted theranostic exosomes. *Biomaterials* 177 139–148. 10.1016/j.biomaterials.2018.05.048 29890363 PMC6019194

[B79] Kapnisi M. Mansfield C. Marijon C. Guex A. G. Perbellini F. Bardi I. (2018). Auxetic cardiac patches with tunable mechanical and conductive properties toward treating myocardial infarction. *Adv. Funct. Mater.* 28:1800618. 10.1002/adfm.201800618 29875619 PMC5985945

[B80] Khan M. Nickoloff E. Abramova T. Johnson J. Verma S. K. Krishnamurthy P. (2015a). Embryonic stem cell–derived exosomes promote endogenous repair mechanisms and enhance cardiac function following myocardial infarction. *Circ. Res.* 117 52–64. 10.1161/CIRCRESAHA.117.305990 25904597 PMC4482130

[B81] Khan M. Xu Y. Hua S. Johnson J. Belevych A. Janssen P. M. L. (2015b). Evaluation of changes in morphology and function of human induced pluripotent stem cell derived cardiomyocytes (HiPSC-CMs) cultured on an aligned-nanofiber cardiac patch. *PLoS One* 10:e0126338. 10.1371/journal.pone.0126338 25993466 PMC4437999

[B82] Kikuchi K. Holdway J. E. Werdich A. A. Anderson R. M. Fang Y. Egnaczyk G. F. (2010). Primary contribution to zebrafish heart regeneration by gata4+ cardiomyocytes. *Nature* 464 601–605. 10.1038/nature08804 20336144 PMC3040215

[B83] Kishore R. Khan M. (2016). More than tiny sacks: stem cell exosomes as cell-free modality for cardiac repair. *Circ. Res.* 118 330–343. 10.1161/CIRCRESAHA.115.307654 26838317 PMC4743531

[B84] Korin E. Bejerano T. Cohen S. (2017). GalNAc bio-functionalization of nanoparticles assembled by electrostatic interactions improves siRNA targeting to the liver. *J. Control. Release* 266 310–320. 10.1016/j.jconrel.2017.10.001 28987883

[B85] Koseva N. S. Rydz J. Stoyanova E. V. Mitova V. A. (2015). “Chapter three - hybrid protein–synthetic polymer nanoparticles for drug delivery,” in *Protein and Peptide Nanoparticles for Drug Delivery*, eds Koseva N. S. Rydz J. Stoyanova E. V. Mitova V. A. (Cambridge, MA: Academic Press), 93–119. 10.1016/bs.apcsb.2014.12.003 25819277

[B86] Laflamme M. A. Murry C. E. (2005). Regenerating the heart. *Nat. Biotechnol.* 23 845–856. 10.1038/nbt1117 16003373

[B87] Laflamme M. A. Murry C. E. (2011). Heart regeneration. *Nature* 473 326–335. 10.1038/nature10147 21593865 PMC4091722

[B88] Lai E. C. (2002). Micro RNAs are complementary to 3’ UTR sequence motifs that mediate negative post-transcriptional regulation. *Nat. Genet.* 30 363–364. 10.1038/ng865 11896390

[B89] Landa N. Miller L. Feinberg M. S. Holbova R. Shachar M. Freeman I. (2008). Effect of injectable alginate implant on cardiac remodeling and function after recent and old infarcts in rat. *Circulation* 117 1388–1396. 10.1161/CIRCULATIONAHA.107.727420 18316487

[B90] Langer R. Vacanti J. P. (1993). Tissue Engineering. *Science* 260 920–926. 10.1126/science.8493529 8493529

[B91] Langer R. S. Peppas N. A. (1981). Present and future applications of biomaterials in controlled drug delivery systems. *Biomaterials* 2 201–214. 10.1016/0142-9612(81)90059-4 7034798

[B92] Lanza R. Langer R. Vacanti J. B. (2014). *Principles of Tissue Engineering*, 4th Edn Boston, MA: Academic Press 10.1016/B978-0-12-398358-9.12001-4

[B93] Leask A. (2007). TGFβ, cardiac fibroblasts, and the fibrotic response. *Cardiovasc. Res.* 74 207–212. 10.1016/j.cardiores.2006.07.012 16919613

[B94] Lee A. Hudson A. R. Shiwarski D. J. Tashman J. W. Hinton T. J. Yerneni S. (2019). 3D bioprinting of collagen to rebuild components of the human heart. *Science* 365 482–487. 10.1126/science.aav9051 31371612

[B95] Lee L. C. Wall S. T. Klepach D. Ge L. Zhang Z. Lee R. J. (2013). Algisyl-LVRTM with coronary artery bypass grafting reduces left ventricular wall stress and improves function in the failing human heart. *Int. J. Cardiol.* 168 2022–2028. 10.1016/j.ijcard.2013.01.003 23394895 PMC3748222

[B96] Lee W. H. Chen W. Shao N. Xiao D. Qin X. Baker N. (2017). Comparison of non-coding RNAs in exosomes and functional efficacy of human embryonic stem cell-versus induced pluripotent stem cell-derived cardiomyocytes. *Stem Cells* 35 2138–2149. 10.1002/stem.2669 28710827 PMC5918285

[B97] Leor J. Aboulafia-Etzion S. Dar A. Shapiro L. Barbash I. M. Battler A. (2000). Bioengineered cardiac grafts: a new approach to repair the infarcted myocardium? *Circulation* 102 Iii–56. 10.1161/01.cir.102.suppl_3.iii-56 11082363

[B98] Leor J. Tuvia S. Guetta V. Manczur F. Castel D. Willenz U. (2009). Intracoronary injection of in situ forming alginate hydrogel reverses left ventricular remodeling after myocardial infarction in swine. *J. Am. Coll. Cardiol.* 54 1014–1023. 10.1016/j.jacc.2009.06.010 19729119

[B99] Levit R. D. Landázuri N. Phelps E. A. Brown M. E. García A. J. Davis M. E. (2013). Cellular encapsulation enhances cardiac repair. *J. Am. Heart Assoc.* 2:e000367. 10.1161/JAHA.113.000367 24113327 PMC3835246

[B100] Li F. Wang X. Capasso J. M. Gerdes A. M. (1996). Rapid transition of cardiac myocytes from hyperplasia to hypertrophy during postnatal development. *J. Mol. Cell. Cardiol.* 28 1737–1746. 10.1006/jmcc.1996.0163 8877783

[B101] Lian X. Zhang J. Azarin S. M. Zhu K. Hazeltine L. B. Bao X. (2013). Directed cardiomyocyte differentiation from human pluripotent stem cells by modulating Wnt/β-catenin signaling under fully defined conditions. *Nat. Protoc.* 8 162–175. 10.1038/nprot.2012.150 23257984 PMC3612968

[B102] Liau B. Christoforou N. Leong K. W. Bursac N. (2011). Pluripotent stem cell-derived cardiac tissue patch with advanced structure and function. *Biomaterials* 32 9180–9187. 10.1016/j.biomaterials.2011.08.050 21906802 PMC3190071

[B103] Liem A. L. van‘t Hof A. W. J. Hoorntje J. C. A. de Boer M.-J. Suryapranata H. Zijlstra F. (1998). Influence of treatment delay on infarct size and clinical outcome in patients with acute myocardial infarction treated with primary angioplasty. *J. Am. Coll. Cardiol.* 32 629–633. 10.1016/s0735-1097(98)00280-0 9741503

[B104] Ling G. Gregorich Z. R. Wuqiang Z. Saidulu M. Yasin O. Xi L. (2018). Large cardiac muscle patches engineered from human induced-pluripotent stem cell–derived cardiac cells improve recovery from myocardial infarction in swine. *Circulation* 137 1712–1730. 10.1161/CIRCULATIONAHA.117.030785 29233823 PMC5903991

[B105] Liu B. Lee B. W. Nakanishi K. Villasante A. Williamson R. Metz J. (2018). Cardiac recovery via extended cell-free delivery of extracellular vesicles secreted by cardiomyocytes derived from induced pluripotent stem cells. *Nat. Biomed. Eng.* 2 293–303. 10.1038/s41551-018-0229-7 30271672 PMC6159913

[B106] Liu X. Ding Y. Zhao B. Liu Y. Luo S. Wu J. (2016). In vitro and in vivo evaluation of puerarin-loaded PEGylated mesoporous silica nanoparticles. *Drug Dev. Ind. Pharm.* 42 2031–2037. 10.1080/03639045.2016.1190742 27282345

[B107] Liu Z. Wang H. Wang Y. Lin Q. Yao A. Cao F. (2012). The influence of chitosan hydrogel on stem cell engraftment, survival and homing in the ischemic myocardial microenvironment. *Biomaterials* 33 3093–3106. 10.1016/j.biomaterials.2011.12.044 22265788

[B108] Lovric J. Mano M. Zentilin L. Eulalio A. Zacchigna S. Giacca M. (2012). Terminal differentiation of cardiac and skeletal myocytes induces permissivity to AAV transduction by relieving inhibition imposed by DNA damage response proteins. *Mol. Ther.* 20 2087–2097. 10.1038/mt.2012.144 22850678 PMC3493462

[B109] Luan X. Sansanaphongpricha K. Myers I. Chen H. Yuan H. Sun D. (2017). Engineering exosomes as refined biological nanoplatforms for drug delivery. *Acta Pharmacol. Sin.* 38 754–763. 10.1038/aps.2017.12 28392567 PMC5520184

[B110] Lutolf M. P. Hubbell J. A. (2005). Synthetic biomaterials as instructive extracellular microenvironments for morphogenesis in tissue engineering. *Nat. Biotechnol.* 23 47–55. 10.1038/nbt1055 15637621

[B111] Malki M. Fleischer S. Shapira A. Dvir T. (2018). Gold nanorod-based engineered cardiac patch for suture-free engraftment by near IR. *Nano Lett.* 18 4069–4073. 10.1021/acs.nanolett.7b04924 29406721 PMC6047511

[B112] Malliaras K. Li T.-S. Luthringer D. Terrovitis J. Cheng K. Chakravarty T. (2012). Safety and efficacy of allogeneic cell therapy in infarcted rats transplanted with mismatched cardiosphere-derived cells. *Circulation* 125 100–112. 10.1161/CIRCULATIONAHA.111.042598 22086878 PMC3256094

[B113] Margolis G. Polyak B. Cohen S. (2018). Magnetic induction of multiscale anisotropy in macroporous alginate scaffolds. *Nano Lett.* 18 7314–7322. 10.1021/acs.nanolett.8b03514 30380888

[B114] Mariani E. Lisignoli G. Borzì R. M. Pulsatelli L. (2019). Biomaterials: foreign bodies or tuners for the immune response? *Int. J. Mol. Sci.* 20:E636. 10.3390/ijms20030636 30717232 PMC6386828

[B115] Matsumura Y. Zhu Y. Jiang H. D’Amore A. Luketich S. K. Charwat V. (2019). Intramyocardial injection of a fully synthetic hydrogel attenuates left ventricular remodeling post myocardial infarction. *Biomaterials* 217:119289. 10.1016/j.biomaterials.2019.119289 31254935

[B116] Matz D. G. Oberpriller J. O. Oberpriller J. C. (1998). Comparison of mitosis in binucleated and mononucleated newt cardiac myocytes. *Anat. Rec.* 251 245–255. 10.1002/(sici)1097-0185(199806)251:2<245::aid-ar14>3.0.co;2-o 9624456

[B117] Mawad D. Mansfield C. Lauto A. Perbellini F. Nelson G. W. Tonkin J. (2016). A conducting polymer with enhanced electronic stability applied in cardiac models. *Sci. Adv.* 2:e1601007. 10.1126/sciadv.1601007 28138526 PMC5262463

[B118] McLaughlin S. McNeill B. Podrebarac J. Hosoyama K. Sedlakova V. Cron G. (2019). Injectable human recombinant collagen matrices limit adverse remodeling and improve cardiac function after myocardial infarction. *Nat. Commun.* 10:4866. 10.1038/s41467-019-12748-8 31653830 PMC6814728

[B119] Menasché P. (2008). Current status and future prospects for cell transplantation to prevent congestive heart failure. *Semin. Thorac. Cardiovasc. Surg.* 20 131–137. 10.1053/j.semtcvs.2008.03.001 18707646

[B120] Menasché P. (2018). Cell therapy trials for heart regeneration—lessons learned and future directions. *Nat. Rev. Cardiol.* 15 659–671. 10.1038/s41569-018-0013-0 29743563

[B121] Menasché P. Vanneaux V. Fabreguettes J.-R. Bel A. Tosca L. Garcia S. (2014). Towards a clinical use of human embryonic stem cell-derived cardiac progenitors: a translational experience. *Eur. Heart J.* 36 743–750. 10.1093/eurheartj/ehu192 24835485

[B122] Menasché P. Vanneaux V. Hagège A. Bel A. Cholley B. Parouchev A. (2018). Transplantation of human embryonic stem cell–derived cardiovascular progenitors for severe ischemic left ventricular dysfunction. *J. Am. Coll. Cardiol.* 71 429–438. 10.1016/j.jacc.2017.11.047 29389360

[B123] Mercola M. Ruiz-Lozano P. Schneider M. D. (2011). Cardiac muscle regeneration: lessons from development. *Genes Dev.* 25 299–309. 10.1101/gad.2018411 21325131 PMC3042154

[B124] Mewhort H. E. M. Turnbull J. D. Satriano A. Chow K. Flewitt J. A. Andrei A.-C. (2016). Epicardial infarct repair with bioinductive extracellular matrix promotes vasculogenesis and myocardial recovery. *J. Hear. Lung Transplant.* 35 661–670. 10.1016/j.healun.2016.01.012 26987597

[B125] Miyagi Y. Chiu L. L. Y. Cimini M. Weisel R. D. Radisic M. Li R.-K. (2011). Biodegradable collagen patch with covalently immobilized VEGF for myocardial repair. *Biomaterials* 32 1280–1290. 10.1016/j.biomaterials.2010.10.007 21035179

[B126] Mohamed T. M. A. Ang Y.-S. Radzinsky E. Zhou P. Huang Y. Elfenbein A. (2018). Regulation of cell cycle to stimulate adult cardiomyocyte proliferation and cardiac regeneration. *Cell* 173 104–116. 10.1016/j.cell.2018.02.014 29502971 PMC5973786

[B127] Montgomery R. L. Hullinger T. G. Semus H. M. Dickinson B. A. Seto A. G. Lynch J. M. (2011). Therapeutic inhibition of miR-208a improves cardiac function and survival during heart failure. *Circulation* 124 1537–1547. 10.1161/CIRCULATIONAHA.111.030932 21900086 PMC3353551

[B128] Mosala Nezhad Z. Poncelet A. de Kerchove L. Gianello P. Fervaille C. El Khoury G. (2016). Small intestinal submucosa extracellular matrix (CorMatrix^®^) in cardiovascular surgery: a systematic review. *Interact. Cardiovasc. Thorac. Surg.* 22 839–850. 10.1093/icvts/ivw020 26912574 PMC4986773

[B129] Muschler G. F. Nakamoto C. Griffith L. G. (2004). Engineering principles of clinical cell-based tissue engineering. *JBJS* 86 1541–1558. 10.2106/00004623-200407000-00029 15252108

[B130] Nelson D. M. Ma Z. Fujimoto K. L. Hashizume R. Wagner W. R. (2011). Intra-myocardial biomaterial injection therapy in the treatment of heart failure: materials, outcomes and challenges. *Acta Biomater.* 7 1–15. 10.1016/j.actbio.2010.06.039 20619368 PMC3208237

[B131] Nelson J. S. Heider A. Si M.-S. Ohye R. G. (2016). Evaluation of explanted CorMatrix intracardiac patches in children with congenital heart disease. *Ann. Thorac. Surg.* 102 1329–1335. 10.1016/j.athoracsur.2016.03.086 27237540

[B132] Nguyen M. M. Carlini A. S. Chien M. Sonnenberg S. Luo C. Braden R. L. (2015). Enzyme-responsive nanoparticles for targeted accumulation and prolonged retention in heart tissue after myocardial infarction. *Adv. Mater.* 27 5547–5552. 10.1002/adma.201502003 26305446 PMC4699559

[B133] Nussbaum J. Minami E. Laflamme M. A. Virag J. A. I. Ware C. B. Masino A. (2007). Transplantation of undifferentiated murine embryonic stem cells in the heart: teratoma formation and immune response. *FASEB J.* 21 1345–1357. 10.1096/fj.06-6769com 17284483

[B134] O’Neill H. S. Gallagher L. B. O’Sullivan J. Whyte W. Curley C. Dolan E. (2016). Biomaterial-enhanced cell and drug delivery: lessons learned in the cardiac field and future perspectives. *Adv. Mater.* 28 5648–5661. 10.1002/adma.201505349 26840955

[B135] Ong S.-B. Hernández-Reséndiz S. Crespo-Avilan G. E. Mukhametshina R. T. Kwek X.-Y. Cabrera-Fuentes H. A. (2018). Inflammation following acute myocardial infarction: multiple players, dynamic roles, and novel therapeutic opportunities. *Pharmacol. Ther.* 186 73–87. 10.1016/j.pharmthera.2018.01.001 29330085 PMC5981007

[B136] Orr S. Strominger I. Eremenko E. Vinogradov E. Ruvinov E. Monsonego A. (2016). TGF-β affinity-bound to a macroporous alginate scaffold generates local and peripheral immunotolerant responses and improves allocell transplantation. *Acta Biomater.* 45 196–209. 10.1016/j.actbio.2016.08.015 27523029

[B137] Parrag I. C. Zandstra P. W. Woodhouse K. A. (2012). Fiber alignment and coculture with fibroblasts improves the differentiated phenotype of murine embryonic stem cell-derived cardiomyocytes for cardiac tissue engineering. *Biotechnol. Bioeng.* 109 813–822. 10.1002/bit.23353 22006660

[B138] Passier R. Oostwaard D. W. Snapper J. Kloots J. Hassink R. J. Kuijk E. (2005). Increased cardiomyocyte differentiation from human embryonic stem cells in serum-free cultures. *Stem Cells* 23 772–780. 10.1634/stemcells.2004-0184 15917473

[B139] Pierluigi L. Giulia P. Valentina M. Gianfranco S. Serena Z. Mauro G. (2017). Single-dose intracardiac injection of pro-regenerative microRNAS improves cardiac function after myocardial infarction. *Circ. Res.* 120 1298–1304. 10.1161/CIRCRESAHA.116.309589 28077443

[B140] Pittenger M. F. Martin B. J. (2004). Mesenchymal stem cells and their potential as cardiac therapeutics. *Circ. Res.* 95 9–20. 10.1161/01.RES.0000135902.99383.6f 15242981

[B141] Porrello E. R. Mahmoud A. I. Simpson E. Hill J. A. Richardson J. A. Olson E. N. (2011). Transient regenerative potential of the neonatal mouse heart. *Science* 331 1078–1080. 10.1126/science.1200708 21350179 PMC3099478

[B142] Porrello E. R. Mahmoud A. I. Simpson E. Johnson B. A. Grinsfelder D. Canseco D. (2013). Regulation of neonatal and adult mammalian heart regeneration by the miR-15 family. *Proc. Natl. Acad. Sci. U.S.A.* 110 187–192. 10.1073/pnas.1208863110 23248315 PMC3538265

[B143] Poss K. D. Wilson L. G. Keating M. T. (2002). Heart regeneration in zebrafish. *Science* 298 2188–2190. 10.1126/science.1077857 12481136

[B144] Prabhakaran M. P. Kai D. Ghasemi-Mobarakeh L. Ramakrishna S. (2011). Electrospun biocomposite nanofibrous patch for cardiac tissue engineering. *Biomed. Mater.* 6:055001. 10.1088/1748-6041/6/5/055001 21813957

[B145] Rabea H. Daniela P. Stefanie Z. Ariane F. Wira H. Quan-Fu X. (2013). Inhibition of microRNA-92a protects against ischemia/reperfusion injury in a large-animal model. *Circulation* 128 1066–1075. 10.1161/CIRCULATIONAHA.113.001904 23897866

[B146] Rao S. V. Zeymer U. Douglas P. S. Al-Khalidi H. White J. A. Liu J. (2016). Bioabsorbable intracoronary matrix for prevention of ventricular remodeling after myocardial infarction. *J. Am. Coll. Cardiol.* 68 715–723. 10.1016/j.jacc.2016.05.053 27515331

[B147] Reis L. A. Chiu L. L. Y. Feric N. Fu L. Radisic M. (2016). Biomaterials in myocardial tissue engineering. *J. Tissue Eng. Regen. Med.* 10 11–28. 10.1002/term.1944 25066525 PMC4933503

[B148] Remaut K. Sanders N. N. De Geest B. G. Braeckmans K. Demeester J. De Smedt S. C. (2007). Nucleic acid delivery: where material sciences and bio-sciences meet. *Mater. Sci. Eng. R Rep.* 58 117–161. 10.1016/j.mser.2007.06.001

[B149] Renault M.-A. Losordo D. W. (2007). Therapeutic myocardial angiogenesis. *Microvasc. Res.* 74 159–171. 10.1016/j.mvr.2007.08.005 17950369 PMC2172411

[B150] Riegler J. Tiburcy M. Ebert A. Tzatzalos E. Raaz U. Abilez O. J. (2015). Human engineered heart muscles engraft and survive long term in a rodent myocardial infarction model. *Circ. Res.* 117 720–730. 10.1161/CIRCRESAHA.115.306985 26291556 PMC4679370

[B151] Roche E. T. Hastings C. L. Lewin S. A. Shvartsman D. E. Brudno Y. Vasilyev N. V. (2014). Comparison of biomaterial delivery vehicles for improving acute retention of stem cells in the infarcted heart. *Biomaterials* 35 6850–6858. 10.1016/j.biomaterials.2014.04.114 24862441 PMC4051834

[B152] Rodell C. B. Lee M. E. Wang H. Takebayashi S. Takayama T. Kawamura T. (2016). Injectable shear-thinning hydrogels for minimally invasive delivery to infarcted myocardium to limit left ventricular remodeling. *Circ. Cardiovasc. Interv.* 9:e004058. 27729419 10.1161/CIRCINTERVENTIONS.116.004058 PMC5123705

[B153] Roy S. M. Sahoo S. K. (2015). “Controlled drug delivery: polymeric biomaterials for,” in *Encyclopedia of Biomedical Polymers and Polymeric Biomaterials*, Vol. 11 (Boca Raton, FL: CRC Press), 2135–2146. 10.1081/e-ebpp-120050023

[B154] Ruan J.-L. Tulloch N. L. Razumova M. V. Saiget M. Muskheli V. Pabon L. (2016). Mechanical stress conditioning and electrical stimulation promote contractility and force maturation of induced pluripotent stem cell-derived human cardiac tissue. *Circulation* 134 1557–1567. 10.1161/CIRCULATIONAHA.114.014998 27737958 PMC5123912

[B155] Ruvinov E. Cohen S. (2016). Alginate biomaterial for the treatment of myocardial infarction: progress, translational strategies, and clinical outlook. From ocean algae to patient bedside. *Adv. Drug Deliv. Rev.* 96 54–76. 10.1016/j.addr.2015.04.021 25962984

[B156] Ruvinov E. Freeman I. Fredo R. Cohen S. (2016). Spontaneous coassembly of biologically active nanoparticles via affinity binding of heparin-binding proteins to alginate-sulfate. *Nano Lett.* 16 883–888. 10.1021/acs.nanolett.5b03598 26745552

[B157] Ruvinov E. Harel-Adar T. Cohen S. (2011a). Bioengineering the infarcted heart by applying bio-inspired materials. *J. Cardiovasc. Transl. Res.* 4 559–574. 10.1007/s12265-011-9288-9 21656074

[B158] Ruvinov E. Kryukov O. Forti E. Korin E. Goldstein M. Cohen S. (2015). Calcium–siRNA nanocomplexes: what reversibility is all about. *J. Control. Release* 203 150–160. 10.1016/j.jconrel.2015.02.029 25702963

[B159] Ruvinov E. Leor J. Cohen S. (2010). The effects of controlled HGF delivery from an affinity-binding alginate biomaterial on angiogenesis and blood perfusion in a hindlimb ischemia model. *Biomaterials* 31 4573–4582. 10.1016/j.biomaterials.2010.02.026 20206988

[B160] Ruvinov E. Leor J. Cohen S. (2011b). The promotion of myocardial repair by the sequential delivery of IGF-1 and HGF from an injectable alginate biomaterial in a model of acute myocardial infarction. *Biomaterials* 32 565–578. 10.1016/j.biomaterials.2010.08.097 20889201

[B161] Ruvinov E. Sapir Y. Cohen S. (2012). Cardiac tissue engineering: principles, materials, and applications. *Synth. Lect. Tissue Eng.* 4 1–200. 10.2200/s00437ed1v01y201207tis009

[B162] Sabbah H. N. Wang M. Gupta R. C. Rastogi S. Ilsar I. Sabbah M. S. (2013). Augmentation of left ventricular wall thickness with alginate hydrogel implants improves left ventricular function and prevents progressive remodeling in dogs with chronic heart failure. *JACC Heart Fail.* 1 252–258. 10.1016/j.jchf.2013.02.006 23998003 PMC3756288

[B163] Sachlos E. Czernuszka J. T. (2003). Making tissue engineering scaffolds work. Review: the application of solid freeform fabrication technology to the production of tissue engineering scaffolds. *Eur. Cell. Mater.* 5 39–40. 10.22203/ecm.v005a03 14562270

[B164] Saleh M. Ambrose J. A. (2018). Understanding myocardial infarction. *F1000Res.* 7:F1000 Faculty Rev-1378. 10.12688/f1000research.15096.1 30228871 PMC6124376

[B165] Sapir Y. Cohen S. Friedman G. Polyak B. (2012). The promotion of in vitro vessel-like organization of endothelial cells in magnetically responsive alginate scaffolds. *Biomaterials* 33 4100–4109. 10.1016/j.biomaterials.2012.02.037 22417620 PMC3565424

[B166] Sapir Y. Kryukov O. Cohen S. (2011). Integration of multiple cell-matrix interactions into alginate scaffolds for promoting cardiac tissue regeneration. *Biomaterials* 32 1838–1847. 10.1016/j.biomaterials.2010.11.008 21112626

[B167] Seif-Naraghi S. B. Singelyn J. M. Salvatore M. A. Osborn K. G. Wang J. J. Sampat U. (2013). Safety and efficacy of an injectable extracellular matrix hydrogel for treating myocardial infarction. *Sci. Transl. Med.* 5:173ra25. 10.1126/scitranslmed.3005503 23427245 PMC3848875

[B168] Shachar M. Cohen S. (2003). Cardiac tissue engineering, ex-vivo: design principles in biomaterials and bioreactors. *Heart Fail. Rev.* 8 271–276. 10.1023/A:1024729919743 12878836

[B169] Shadrin I. Y. Allen B. W. Qian Y. Jackman C. P. Carlson A. L. Juhas M. E. (2017). Cardiopatch platform enables maturation and scale-up of human pluripotent stem cell-derived engineered heart tissues. *Nat. Commun.* 8:1825. 10.1038/s41467-017-01946-x 29184059 PMC5705709

[B170] Shapira A. Feiner R. Dvir T. (2016). Composite biomaterial scaffolds for cardiac tissue engineering. *Int. Mater. Rev.* 61 1–19. 10.1179/1743280415y.0000000012

[B171] Shapiro L. Cohen S. (1997). Novel alginate sponges for cell culture and transplantation. *Biomaterials* 18 583–590. 10.1016/S0142-9612(96)00181-0 9134157

[B172] Shevach M. Fleischer S. Shapira A. Dvir T. (2014). Gold nanoparticle-decellularized matrix hybrids for cardiac tissue engineering. *Nano Lett.* 14 5792–5796. 10.1021/nl502673m 25176294

[B173] Shevach M. Zax R. Abrahamov A. Fleischer S. Shapira A. Dvir T. (2015). Omentum ECM-based hydrogel as a platform for cardiac cell delivery. *Biomed. Mater.* 10:34106. 10.1088/1748-6041/10/3/034106 25970726

[B174] Silva G. V. Litovsky S. Assad J. A. R. Sousa A. L. S. Martin B. J. Vela D. (2005). Mesenchymal stem cells differentiate into an endothelial phenotype, enhance vascular density, and improve heart function in a canine chronic ischemia model. *Circulation* 111 150–156. 10.1161/01.CIR.0000151812.86142.45 15642764

[B175] Simons M. Annex B. H. Laham R. J. Kleiman N. Henry T. Dauerman H. (2002). Pharmacological treatment of coronary artery disease with recombinant fibroblast growth factor-2: double-blind, randomized, controlled clinical trial. *Circulation* 105 788–793. 10.1161/hc0802.104407 11854116

[B176] Simons M. Raposo G. (2009). Exosomes–vesicular carriers for intercellular communication. *Curr. Opin. Cell Biol.* 21 575–581. 10.1016/j.ceb.2009.03.007 19442504

[B177] Singh A. Singh A. Sen D. (2016). Mesenchymal stem cells in cardiac regeneration: a detailed progress report of the last 6 years (2010-2015). *Stem Cell Res. Ther.* 7:82. 10.1186/s13287-016-0341-0 27259550 PMC4893234

[B178] Sun D. Zhuang X. Xiang X. Liu Y. Zhang S. Liu C. (2010). A novel nanoparticle drug delivery system: the anti-inflammatory activity of curcumin is enhanced when encapsulated in exosomes. *Mol. Ther.* 18 1606–1614. 10.1038/mt.2010.105 20571541 PMC2956928

[B179] Sutton M. G. S. J. Sharpe N. (2000). Left ventricular remodeling after myocardial infarction: pathophysiology and therapy. *Circulation* 101 2981–2988. 10.1161/01.cir.101.25.2981 10869273

[B180] Tang J. Wang J. Huang K. Ye Y. Su T. Qiao L. (2018). Cardiac cell–integrated microneedle patch for treating myocardial infarction. *Sci. Adv.* 4:eaat9365. 10.1126/sciadv.aat9365 30498778 PMC6261659

[B181] Thakker R. Yang P. (2014). Mesenchymal stem cell therapy for cardiac repair. *Curr. Treat. Options Cardiovasc. Med.* 16:323. 10.1007/s11936-014-0323-4 24898315 PMC4295923

[B182] Tibbitt M. W. Langer R. (2017). Living biomaterials. *Acc Chem. Res.* 50 508–513. 10.1021/acs.accounts.6b00499 28945422

[B183] Torella D. Ellison G. M. Méndez-Ferrer S. Ibanez B. Nadal-Ginard B. (2006). Resident human cardiac stem cells: role in cardiac cellular homeostasis and potential for myocardial regeneration. *Nat. Rev. Cardiol.* 3 S8–S13. 10.1038/ncpcardio0409 16501638

[B184] Torella D. Rota M. Nurzynska D. Musso E. Monsen A. Shiraishi I. (2004). Cardiac stem cell and myocyte aging, heart failure, and insulin-like growth factor-1 overexpression. *Circ. Res.* 94 514–524. 10.1161/01.res.0000117306.10142.50 14726476

[B185] Traverse J. H. Henry T. D. Dib N. Patel A. N. Pepine C. Schaer G. L. (2019). First-in-man study of a cardiac extracellular matrix hydrogel in early and late myocardial infarction patients. *JACC Basic Transl. Sci.* 4 659–669. 10.1016/j.jacbts.2019.07.012 31709316 PMC6834965

[B186] Tsur-Gang O. Ruvinov E. Landa N. Holbova R. Feinberg M. S. Leor J. (2009). The effects of peptide-based modification of alginate on left ventricular remodeling and function after myocardial infarction. *Biomaterials* 30 189–195. 10.1016/j.biomaterials.2008.09.018 18849071

[B187] Urbanek K. Torella D. Sheikh F. De Angelis A. Nurzynska D. Silvestri F. (2005). Myocardial regeneration by activation of multipotent cardiac stem cells in ischemic heart failure. *Proc. Natl. Acad. Sci. U.S.A.* 102 8692–8697. 10.1073/pnas.0500169102 15932947 PMC1150816

[B188] Vagnozzi R. J. Maillet M. Sargent M. A. Khalil H. Johansen A. K. Schwanekamp J. A. (2019). An acute immune response underlies the benefit of cardiac stem-cell therapy. *Nature* 577 405–409. 10.1038/s41586-019-1802-2 31775156 PMC6962570

[B189] Vaishya R. D. Mandal A. Gokulgandhi M. Patel S. Mitra A. K. (2015). Reversible hydrophobic ion-paring complex strategy to minimize acylation of octreotide during long-term delivery from PLGA microparticles. *Int. J. Pharm.* 489 237–245. 10.1016/j.ijpharm.2015.04.075 25940041 PMC4457703

[B190] Vandergriff A. Huang K. Shen D. Hu S. Hensley M. T. Caranasos T. G. (2018). Targeting regenerative exosomes to myocardial infarction using cardiac homing peptide. *Theranostics* 8 1869–1878. 10.7150/thno.20524 29556361 PMC5858505

[B191] Wang L. L. Liu Y. Chung J. J. Wang T. Gaffey A. C. Lu M. (2017). Sustained miRNA delivery from an injectable hydrogel promotes cardiomyocyte proliferation and functional regeneration after ischaemic injury. *Nat. Biomed. Eng.* 1 983–992. 10.1038/s41551-017-0157-y 29354322 PMC5773070

[B192] Wassenaar J. W. Gaetani R. Garcia J. J. Braden R. L. Luo C. G. Huang D. (2016). Evidence for mechanisms underlying the functional benefits of a myocardial matrix hydrogel for post-MI treatment. *J. Am. Coll. Cardiol.* 67 1074–1086. 10.1016/j.jacc.2015.12.035 26940929 PMC4779189

[B193] Webber J. Steadman R. Mason M. D. Tabi Z. Clayton A. (2010). Cancer exosomes trigger fibroblast to myofibroblast differentiation. *Cancer Res.* 70 9621–9630. 10.1158/0008-5472.CAN-10-1722 21098712

[B194] Wehman B. Sharma S. Pietris N. Mishra R. Siddiqui O. T. Bigham G. (2016). Mesenchymal stem cells preserve neonatal right ventricular function in a porcine model of pressure overload. *Am. J. Physiol. Circ. Physiol.* 310 H1816–H1826. 10.1152/ajpheart.00955.2015 27106046

[B195] Wei H.-J. Chen C.-H. Lee W.-Y. Chiu I. Hwang S.-M. Lin W.-W. (2008). Bioengineered cardiac patch constructed from multilayered mesenchymal stem cells for myocardial repair. *Biomaterials* 29 3547–3556. 10.1016/j.biomaterials.2008.05.009 18538386

[B196] Wendel J. S. Ye L. Zhang P. Tranquillo R. T. Zhang J. J. (2013). Functional consequences of a tissue-engineered myocardial patch for cardiac repair in a rat infarct model. *Tissue Eng. Part A* 20 1325–1335. 10.1089/ten.tea.2013.0312 24295499 PMC3993032

[B197] Wong P. T. Choi S. K. (2015). Mechanisms of drug release in nanotherapeutic delivery systems. *Chem. Rev.* 115 3388–3432. 10.1021/cr5004634 25914945

[B198] Wu Y. Wang L. Guo B. Ma P. X. (2017). Interwoven aligned conductive nanofiber yarn/hydrogel composite scaffolds for engineered 3d cardiac anisotropy. *ACS Nano* 11 5646–5659. 10.1021/acsnano.7b01062 28590127

[B199] Yanamandala M. Zhu W. Garry D. J. Kamp T. J. Hare J. M. Jun H. (2017). Overcoming the roadblocks to cardiac cell therapy using tissue engineering. *J. Am. Coll. Cardiol.* 70 766–775. 10.1016/j.jacc.2017.06.012 28774384 PMC5553556

[B200] Yang H. Wei L. Liu C. Zhong W. Li B. Chen Y. (2019). Engineering human ventricular heart tissue based on macroporous iron oxide scaffolds. *Acta Biomater.* 88 540–553. 10.1016/j.actbio.2019.02.024 30779999

[B201] Yang S. Leong K.-F. Du Z. Chua C.-K. (2001). The design of scaffolds for use in tissue engineering. Part I. Traditional factors. *Tissue Eng.* 7 679–689. 10.1089/107632701753337645 11749726

[B202] Yost M. J. Baicu C. F. Stonerock C. E. Goodwin R. L. Price R. L. Davis J. M. (2004). A novel tubular scaffold for cardiovascular tissue engineering. *Tissue Eng.* 10 273–284. 10.1089/107632704322791916 15009952

[B203] Zadpoor A. A. Malda J. (2017). Additive manufacturing of biomaterials, tissues, and organs. *Ann. Biomed. Eng.* 45 1–11. 10.1007/s10439-016-1719-y 27632024

[B204] Zhang D. Lee H. Zhu Z. Minhas J. K. Jin Y. (2016). Enrichment of selective miRNAs in exosomes and delivery of exosomal miRNAs *in vitro* and *in vivo*. *Am. J. Physiol. Circ. Physiol.* 312 L110–L121. 10.1152/ajplung.00423.2016 PMC5283929 27881406

[B205] Zhang D. Shadrin I. Y. Lam J. Xian H.-Q. Snodgrass H. R. Bursac N. (2013). Tissue-engineered cardiac patch for advanced functional maturation of human ESC-derived cardiomyocytes. *Biomaterials* 34 5813–5820. 10.1016/j.biomaterials.2013.04.026 23642535 PMC3660435

[B206] Zhang J. Zhu W. Radisic M. Vunjak-Novakovic G. (2018). Can we engineer a human cardiac patch for therapy? *Circ. Res.* 123 244–265. 10.1161/CIRCRESAHA.118.311213 29976691 PMC7250155

[B207] Zhang L. Qian Z. Tahtinen M. Qi S. Zhao F. (2018). Prevascularization of natural nanofibrous extracellular matrix for engineering completely biological three-dimensional prevascularized tissues for diverse applications. *J. Tissue Eng. Regen. Med.* 12 e1325–e1336. 10.1002/term.2512 28714140 PMC5771986

[B208] Zhang Y. Liu D. Chen X. Li J. Li L. Bian Z. (2010). Secreted monocytic miR-150 enhances targeted endothelial cell migration. *Mol. Cell* 39 133–144. 10.1016/j.molcel.2010.06.010 20603081

